# Respiratory diseases and gut microbiota: relevance, pathogenesis, and treatment

**DOI:** 10.3389/fmicb.2024.1358597

**Published:** 2024-07-16

**Authors:** Mengdi Sun, Fang Lu, Donghua Yu, Yu Wang, Pingping Chen, Shumin Liu

**Affiliations:** ^1^Graduate School, Heilongjiang University of Chinese Medicine, Harbin, China; ^2^Institute of Traditional Chinese Medicine, Heilongjiang University of Chinese Medicine, Harbin, China

**Keywords:** gut-lung axis, microbiota, immunology, respiratory system, diagnosis

## Abstract

Preclinical evidence has firmly established a bidirectional interaction among the lung, gut, and gut microbiome. There are many complex communication pathways between the lung and intestine, which affect each other's balance. Some metabolites produced by intestinal microorganisms, intestinal immune cells, and immune factors enter lung tissue through blood circulation and participate in lung immune function. Altered gut–lung–microbiome interactions have been identified in rodent models and humans of several lung diseases such as pulmonary fibrosis, chronic obstructive pulmonary disease, lung cancer, asthma, etc. Emerging evidence suggests that microbial therapies can prevent and treat respiratory diseases, but it is unclear whether this association is a simple correlation with the pathological mechanisms of the disease or the result of causation. In this review, we summarize the complex and critical link between the gut microbiota and the lung, as well as the influence and mechanism of the gut microbiota on respiratory diseases, and discuss the role of interventions such as prebiotics and fecal bacteria transplantation on respiratory diseases. To provide a reference for the rational design of large-scale clinical studies, the direct application of microbial therapy to respiratory-related diseases can reduce the incidence and severity of diseases and accompanying complications.

## 1 Introduction

At present, despite the progress in diagnosis and treatment, respiratory diseases are still a global problem. Pneumonia and lower respiratory infections are the fourth most common cause of death worldwide, while lung cancer is the sixth most common cause of death (Li Z.-J. et al., 2021). In 2017, the International Respiratory Society Forum identified chronic obstructive pulmonary disease (COPD) as the most common respiratory system disease (Labaki and Han, 2020). According to statistics, 544.9 million people worldwide suffer from chronic respiratory diseases, and the COVID-19 pandemic has affected nearly 671.7 million people worldwide and caused nearly seven million deaths (Chow et al., 2023).

Numerous studies have linked the microbiome and its function to lung diseases (e.g. lung cancer, chronic obstructive pulmonary disease, asthma, acute lower respiratory infection, and tuberculosis) (Enaud et al., 2020). The scientific community once believed that microbes did not exist in the lungs (Dickson et al., 2016). Even when bacterial DNA is detected in the lungs, it is often assumed to be technical contamination (Salter et al., 2014). However, with the development of bacterial culture conditions and next-generation sequencing technology, as well as verification from laboratories around the world, the old view of “lungs are sterile” has been overturned (Wypych et al., 2019).

In addition, it is also influenced by microbial signals from distant sites in the body, such as the gut (Dumas et al., 2018; Aishwarya et al., 2022; Gokulan et al., 2022). For example, SCFAs can induce bone marrow hematopoiesis, stimulate airway immunity, and shape the pulmonary immune microenvironment (Dang and Marsland, 2019). Nowadays, numerous studies on the gut–lung axis have presented new evidence, and studies of microbiota transplantation, microbiota-dependent pathways, and downstream metabolites all suggest that the microbiota may affect host metabolism and respiratory disease through multiple metabolic pathways and suggest early the possibility of intervention not only to modify the disease progression but also to delay or prevent the onset of the disease (Thibeault et al., 2021; de Vos et al., 2022). In this review, we describe the latest progress in gut microbiota–lung research, summarize the relationship between gut microbiota and respiratory diseases, and provide references for the in-depth elaboration of the gut–lung axis theory and the treatment of respiratory diseases.

## 2 Gut–lung axis

**Figure 1:**
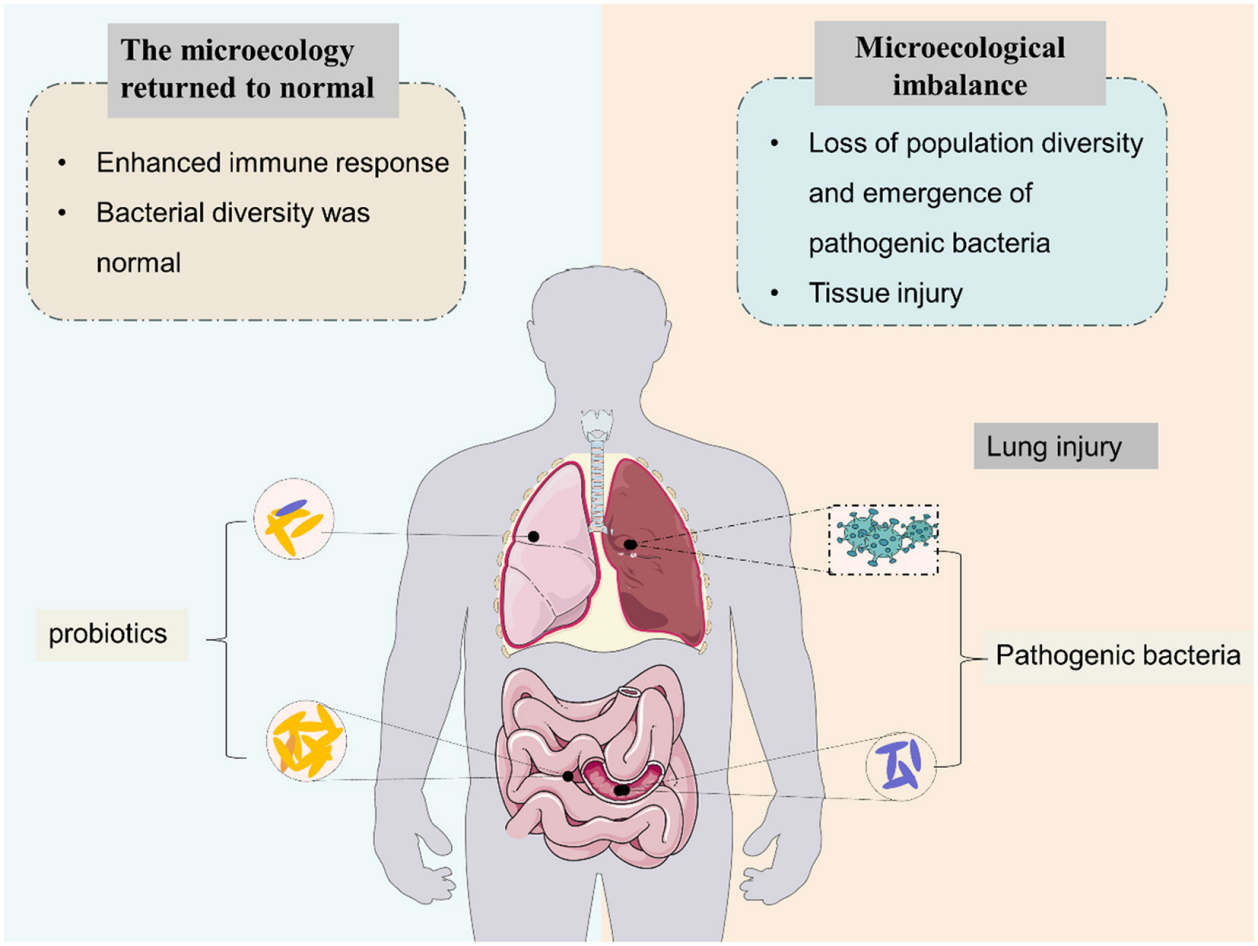
Effects of intestinal microbiota on pulmonary immune microenvironment homeostasis.

The human gut microbiome is a diverse ecosystem that we already know plays an important role in the health of multiple organ systems, and gut dysbiosis may lead to a variety of common diseases such as diabetes, neuropsychiatric disorders, cancer, etc. (Liu et al., 2020; Lau et al., 2021; Bai et al., 2022; Chidambaram et al., 2022). Collectively, the human gut microbiota consists of more than 100 trillion bacteria and more than three million unique genes (Zhuang et al., 2019). The gut microbiota is dominated by *Firmicutes* (e.g. *Lactobacillus, Bacillus*, and *Clostridium*), *Bacteroidetes* (e.g. *Bacteroides*), in addition to *Proteobacteria* (e.g. *Escherichia*) and *Actinobacteria* (e.g. *Bifidobacterium*) (Zhao et al., 2019). Experimental and clinical evidence suggests that the gut microbiota plays a crucial role in maintaining human health. Tightly regulated microbiota–host interactions influence the development, guidance, and priming of the immune system. The mechanisms by which gut microbiota regulate immune responses depend on microbe-associated molecular patterns, microbial metabolites, and interactions of microbes with progenitor and mature immune cells (Sencio et al., 2021). Normal gut flora plays various beneficial roles in the human body, including (a) enhanced immune response and (b) maintaining the intestinal microenvironment in a steady state (Figure 1).

The microbial biomass in the respiratory tract is significantly lower than that in the gut and oral cavity. Due to the lung's long-term exposure to the external environment and its unique topology, the pulmonary flora is in a dynamic state of change, which is mainly shaped by three factors: (1) microbial immigration, (2) microbial emigration or elimination by the immune system, and (3) microbial replication (Whiteside et al., 2021). *Firmicutes* and *Bacteroidetes* are the main groups in the healthy lung microbiome, with *Prevotella, Vesteria*, and *Streptococcus* being the most common (Yagi et al., 2021). In the healthy state of the lungs, there is a steady balance of microorganisms “moving in and out” (Wu and Segal, 2017). The source of the airway microbiome is the upper airway microbiome migrates into the lungs through “micro-respiration,” which occurs mainly during sleep. At the same time, host defense mechanisms are involved in microbial clearance, such as alveolar macrophage clearance and mucociliary transport (Elgamal et al., 2021). When the lung is sick, when the balance of the airway flora is also disrupted, and bacterial proliferation seems to outstrip the ability of the airways to clear microorganisms, the cilia of the lungs are dysfunctional, mucus secretion is increased, and bacterial migration is increased, resulting in increased microbial density, some of which may come from gut microbes, for example, gastroesophageal reflux (Willers and Viemann, 2021).

**Figure 2:**
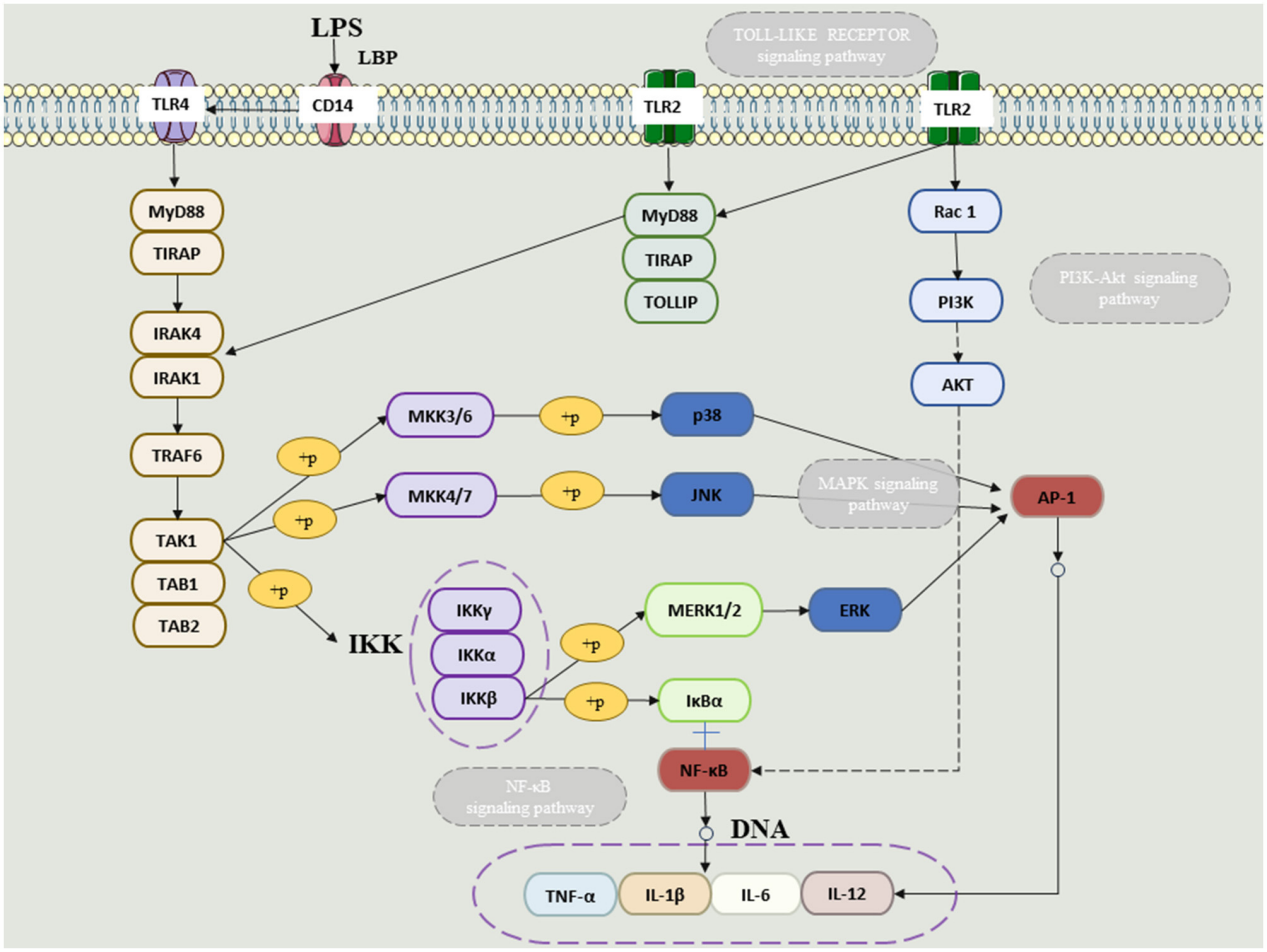
The NFKB and MAPkinase (ERK, JUNK and P38) linked to TLR receptors in the presence of LPS.

“Gut–microbiome–lung” is one of the important areas of modern microbiome research. Gut microbiota disturbances are associated with increased lung inflammation; lung infection, in turn, exacerbates gut dysbiosis. Therefore, based on the “lung–intestinal axis” theory, this paper aims to clarify that microbial therapy should be another important research direction and entry point for the treatment of respiratory diseases in the future by elaborating on the lung–intestinal relationship and provides a more adequate theoretical basis for the clinical treatment of respiratory diseases (Figure 2).

## 3 The main communication pathway between the “gut–lung axis”

### 3.1 Soluble components or metabolites of gut microbiota affect lung diseases through immune regulation

The gut microbiome through anaerobic fermentation of dietary fibers such as resistant starch, pectin, and cellulose produces a multitude of metabolites, particularly SCFAs (Parada Venegas et al., 2019). SCFAs are anti-inflammatory chemicals with immunomodulatory functions, including inhibiting chemotaxis and adhesion of immune cells, as well as inducing the expression of anti-inflammatory cytokines and stimulating apoptosis of immune cells (Ratajczak et al., 2019). Patients with digestive problems have lower levels of SCFA and are more likely to develop lung disease (Dang and Marsland, 2019). It is well known that short-chain fatty acids will produce SCFAs in the gut. The basic role of SCFAs is to reduce intestinal pH and promote mucin synthesis, thereby preventing the development and adhesion of harmful bacteria, enhancing the integrity of epithelial cells, and enhancing host system immunity (Fukuda et al., 2011). SCFAs may increase the number and function of T regulatory (Treg), T helper (Th) 1, and Th17 effector cells by inhibiting histone deacetylases (HDACs), thereby reducing excessive inflammation and immune responses in gastrointestinal and airway diseases (Li et al., 2018). SCFAs can enter the body circulation through passive diffusion and active transport, the latter mediated primarily by transporters such as monocarboxylate transporters 1 and 4 (MCT1, MCT4) and sodium-bound monocarboxylate transporters 1 and 2 (SMCT1, SCMT2) (Sivaprakasam et al., 2017; Ranjbar et al., 2021). SCFAs have been shown to interact with different receptors, including g protein-coupled receptors (GPCRs) GPR43 and GPR41 (Sturm et al., 2021). Immune cells, including lymphocytes, monocytes, and neutrophils, exhibit GPR43 specifically (Le Poul et al., 2003), while GPR41 is expressed more widely in many tissues, including adipose tissue, pancreas, spleen, and lymph nodes, and different SCFAs have different affinities to the two receptors (Li et al., 2018). Another important biological role of SCFAs is that of HDAC inhibitors, particularly by butyric acid (Chang et al., 2014). HDAC inhibition in turn suppresses lipopolysaccharide (LPS)-induced nitric oxide synthesis via inducible nitric oxide synthase (iNOS) and the release of LPS-induced proinflammatory cytokines (IL-6, IL-12) (He et al., 2020). In addition, the anti-inflammatory effect of butyrate is associated with reduced activity of the NF-κB signaling pathway, while stimulating monocytes such as neutrophils to produce anti-inflammatory cytokines such as IL-10 (Ranjbar et al., 2021). SCFAs have anti-inflammatory and immunomodulatory properties and are involved in the recruitment, differentiation, and activation of neutrophils, dendritic cells (DCs), monocytes, macrophages, and T cells. SCFAs inhibit the maturation of dendritic cells, macrophages, and monocytes by inhibiting their ability to recognize antigens and produce pro-inflammatory cytokines such as IL-12 and TNF-α (Corrêa-Oliveira et al., 2016). Further studies have shown that SCFAs can have either anti-inflammatory or pro-inflammatory effects on lung cells, depending on the cell type or SCFA concentration tested (Corrêa-Oliveira et al., 2016). This suggests that, depending on the cell type and specific chemical environment, SCFAs can act as pro-inflammatory or anti-inflammatory chemicals. Not only that, SCFA synthesis induced by high-fiber intake has been shown to influence bone marrow hematopoiesis, speeding up the production of macrophages and DC precursors, and then seeding the lungs with highly phagocytic DCs (Dang and Marsland, 2019). By reducing immune cell migration and proliferation of various cytokines, and causing cell apoptosis, short-chain fatty acids can reduce inflammation (Liu et al., 2009). As a result, SCFAs have powerful anti-inflammatory properties (Figure 2).

### 3.2 Lung and gut microbes interact with each other by altering the body's immune system

There is a migration of immune mediators between the gut and respiratory microbiota, which allows the immune systems of the two sites to interact with each other, creating a synergistic effect. On the one hand, immune cells can migrate. Because of the structural and functional similarities between intestinal mucosa and airway mucosa, they express common homing chemical factor receptors such as chemokine ligand 28 (CCL28) and chemokine receptor 9 (CCR9). Depending on these receptors, lymphocytes can migrate between the intestine and the airway through peripheral blood circulation (Velikova et al., 2021). Gut-associated lymphoid tissue (GALT) is an important link between the lung and the intestine, which plays a key role in inducing immunity and controlling the communication between intestinal mucosa and systemic immunity (Mörbe et al., 2021). B cells in GALT produce antibodies, and T cells target viral or fungus-infected cells, cancer cells, and transplanted cells, all of which contribute to the body's immune response, which in turn affects the respiratory system (Kato et al., 2013; Lacorcia et al., 2021). On the other hand, due to the memory properties of immune cells, intestinal immune cells may develop immune memory after interacting with intestinal microorganisms, making them more flexible in responding to respiratory pathogens. Changes in systemic immune mechanisms are achieved through DCs, which recognize pathogen-associated molecular patterns, migrate to mesenteric lymph nodes and prime T cells, activate various T cell subsets, and regulate the production of cytokines, such as IL 10, TGF-β, INF-γ, and IL 6, T cell subsets then acquire immune homing receptors, such as CC chemokine receptor type 4, resulting in migration to different areas, including the respiratory mucosa, thereby promoting protective and anti-inflammatory response at specific sites (Allard et al., 2018). Relevant immune cells involved in the immune process of the “gut–lung axis” include Th17 cells, Treg cells, invariant natural killer T cells (iNKT), etc. (Tulic et al., 2016).

Th17 may be an important correlate of the “lung–gut axis.” It is important for maintaining mucosal barrier function and clearance of pathogens, and its imbalance is associated with a variety of inflammatory diseases. Colonization of segmented filamentous bacteria (SFB) in the intestinal microbiota can inhibit the proliferation of *Staphylococcus aureus* and *Aspergillus fumigatus* in the lungs by activating Th17 cells and IL-22 (Gauguet et al., 2015). Further studies found that in autoimmune-susceptible mice, SFB-induced Th17 cells can be recruited to the lungs by chemokine ligand 20, leading to autoimmune lung disease and respiratory infections that induce Th17 responses may lead to intestinal damage (Bradley et al., 2017). Gut-derived immune responses can influence lung inflammation, and respiratory infections can also lead to enteritis, suggesting a lung–gut interaction.

Tregs play a crucial role in immune homeostasis. Gut microbiome and their metabolites can regulate Treg differentiation. Tregs regulate the production of the main mucosal antibody immunoglobin A (IgA) through the production of TGF-β, thereby reducing the systemic inflammation caused by the global activation of CD4^+^T cells. Respiratory diseases may occur when IgA response is impaired (Kageyama et al., 2024).

**Figure 3:**
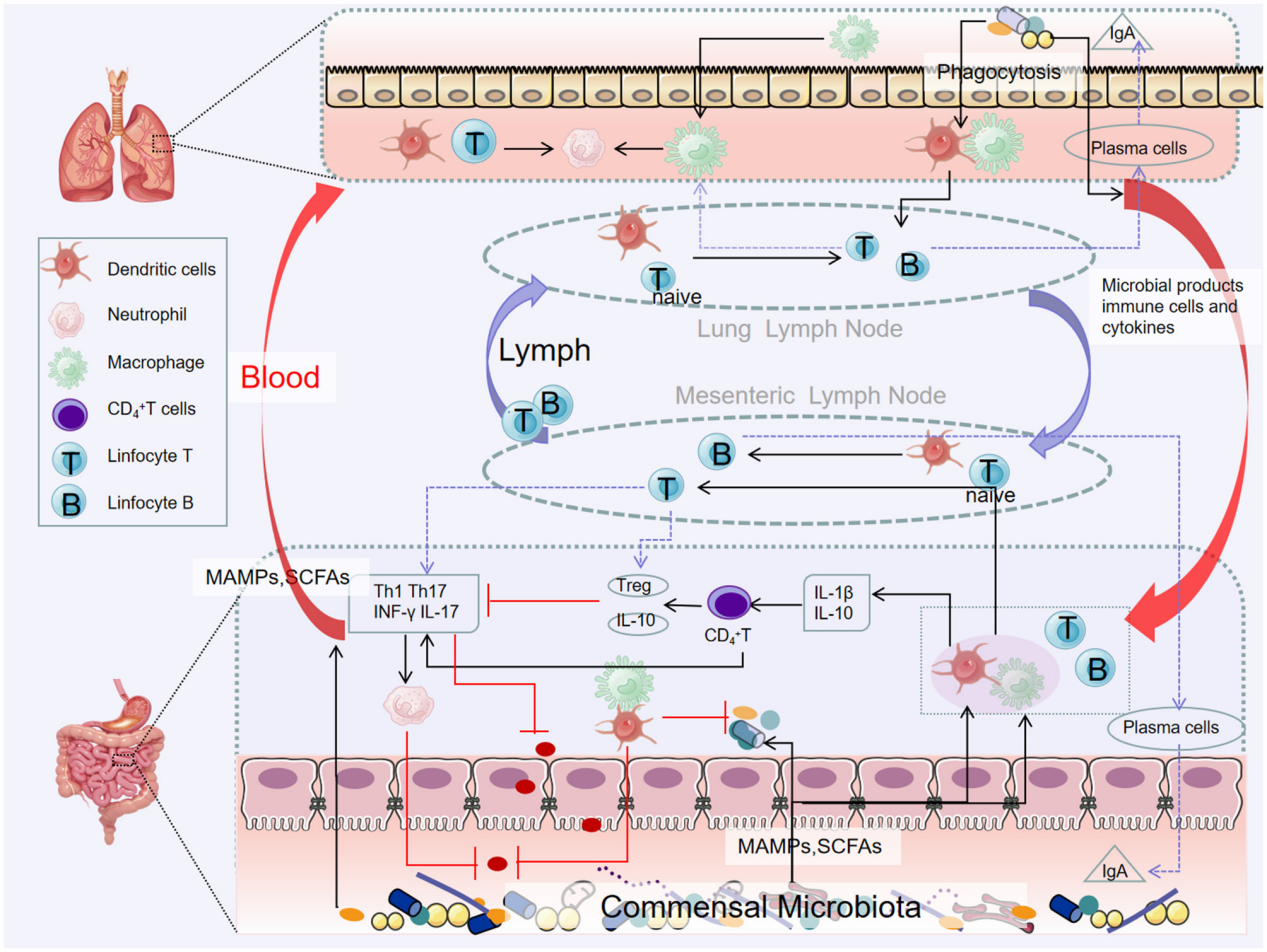
Pathways of the gut–lung axis interaction. Microbial products and metabolites or surviving bacteria propagate from the intestinal mucosa to the lungs, systemic circulation through the lymphatic and blood circulation. Certain microorganisms produce large amounts of short-chain fatty acids when they metabolize dietary fiber. Short-chain fatty acids can be transported to different parts through the blood circulation to affect the recruitment and activity of immune cells in the body (including the lungs), thereby reducing inflammation.

iNKT connects innate and adaptive immunity and can release a large number of cytokines, mainly Th1 or Th2, whose dysfunction is associated with a variety of inflammatory diseases (Jeong et al., 2023). In germ-free mice, iNKT cells accumulate and inflammatory response is enhanced, and the recovery of the intestinal microbiome in the neonatal period can limit the inflammatory trend, suggesting that the microbiome plays a key role in iNKT development, and the intestinal microbiome has the function of directing pulmonary iNKT differentiation (Olszak et al., 2012) (Figure 3).

## 4 Possible mechanisms of gut microbiota in respiratory diseases

### 4.1 Idiopathic pulmonary fibrosis

Idiopathic pulmonary fibrosis (IPF) is a chronic progressive fibrotic interstitial pneumonia of unknown etiology that mainly occurs in the elderly (Ma et al., 2022). IPF has a median survival of 3–5 years after diagnosis and is characterized by inflammation and excessive deposition of extracellular matrix (ECM) in the lungs (Mei et al., 2022), leading to structural changes in the lung parenchyma, such as lung wall thickening and stiffness, which may eventually lead to acute decreased respiratory function and organ failure; high morbidity and mortality due to lung injury from IPF is often progressive and irreversible. It can be diagnosed by chest high-resolution computed tomography. The incidence is increasing year by year, and the treatment is limited (Janowiak et al., 2022). In recent years, some epidemiological and experimental studies have found a direct link between the gut microbiome and pulmonary fibrosis. Changes in intestinal flora and its metabolites can activate a variety of immune cells and non-immune cells of the host, induce an inflammatory response, and trigger mesenchymal cells to produce a large number of ECM components, which is a common cause of fibrotic lesions in other distal extrenteral organs (Zhan et al., 2021). Therefore, it is essential to explore and determine the pathogenesis and immune regulation of pulmonary fibrosis diseases for exploring new therapeutic strategies and therapeutic targets for this disease.

Zhou et al. (2019) used 16S rRNA gene sequencing to compare the intestinal microbiota composition of stool samples from 18 patients with silica-induced pulmonary fibrosis and 21 healthy subjects, and the results showed that the intestinal microbiota of patients with pulmonary fibrosis had significant changes; the levels of *Firmicutes, Actinobacteria, Devosia*, Clostridiales, *Alloprevotella*, and *Rikenellaceae_RC9* were significantly decreased. Animal studies have shown that intestinal flora disturbance caused by antibiotic exposure in young mice can promote the formation of skin and lung fibrosis symptoms in adult mice (Ho and Varga, 2017). The study on the mouse model of idiopathic pulmonary fibrosis found that intestinal bacteria of 412 genera had significant changes with 26 kinds of metabolites. For example, the abundance of Helicobacter was significantly down, while the abundance of *Dubosiella* was significantly increased, and the abundance of *Dubosiella* was significantly positively correlated with the level of betaine in serum. Therefore, this study is the first to validate the possibility and reliability of using gut microbiota and metabolites as biomarkers of the fibrosis process (Gong et al., 2021). In addition, *in vitro* experiments found that SCFA butyrate (C4) produced by gut microbiome metabolism can prevent TGF-β1-induced differentiation of MRC5 human fetal lung fibroblasts into myofibroblasts, inhibit the expression of fibrosis markers, and enhance mitochondrial function, exhibiting potent anti-fibrotic activity (Lee et al., 2021).

Although the sample size of these experiments is small, they may still be helpful for the early diagnosis of pulmonary fibrosis disease and the exploration of an intervention program to delay or inhibit pulmonary fibrosis. However, the causal relationship between the differences in gut microbiota and the progression of pulmonary fibrosis requires further mechanistic studies. Like many studies of gut microbiota, it is difficult to distinguish pathogenic factors from associated effects, and whether different gut microbiota are merely intestinal phenotypes of PF or can influence each other remains unknown. Whether the changes in the intestinal barrier, the exudation of intestinal bacterial metabolites affect the PF or the immune response generated by PF connects the lung to the gut needs to be further explored.

### 4.2 Chronic obstructive pulmonary disease

Chronic obstructive pulmonary disease (COPD), affecting more than 500 million people worldwide, is characterized by dyspnea, chronic cough, and increased sputum, which is associated with severe lung damage from irreversible progressive inflammation and airflow limitation (Vogelmeier et al., 2020). The classification of COPD severity includes four stages based on spirometry and the severity of airflow limitation from stage I to stage IV (Fazleen and Wilkinson, 2020). The mechanism of occurrence and development is still largely unknown, genetic factors, inflammation, immune response, and other factors are reflected in the pathogenesis, it has many comorbidities, such as cardiovascular disease, colitis, and osteoporosis (Ekbom et al., 2008; Li Y. et al., 2022; Polman et al., 2024). It is the third leading cause of death worldwide, with major risk factors being inhalation of cigarette smoke, air pollution, or other toxic substances (O'Donnell et al., 2020). Studies have found that patients with COPD often have gastrointestinal symptoms, which are highly correlated with the severity of the disease (De Nuccio et al., 2022). The imbalance of intestinal microbial ecology can lead to respiratory epithelial damage, lung innate immune defense damage, and harmful bacterial colonization, causing lung inflammation and further aggravating COPD.

Bowerman et al. (2020) compared the gut microbiota of COPD patients and healthy controls and found that the abundance of *Streptococcus, Rothia, Romboutsia, Intestinibacter*, and *Escherichia* increased in COPD, but that of *Bacteroides, Roseburia*, and *Lachnospira* had reduced. Several species of *Streptococcus* and *Lachnospiraceae* also correlated with reduced lung function. In another study, Chiu et al. (2021) investigated how the gut microbiome varies depending on the severity of COPD. The data showed that in patients with severe COPD, the genera *Aerococcus* and *Fusobacterium* were the most abundant, while the contents of *Ruminococcaceae* and *Lachnoclostridium* were low. Jiao et al. (2022) conducted a study on COPD rats, and the results showed that the onset and development of COPD were associated with the imbalance of 41 differential metabolites in plasma, bronchoalveolar lavage fluid and feces, and 82 bacteria at the levels of phylum, class, order, family, and genus from lung and intestine, including *Escherichia-Shigella*. In addition, Wang et al. (2024) found that down-regulating levels of IFN-γ, TNF-α, and IL-6, and restoring the abundance of *Akkermansia* and *Escherichia_Shigella* improved LPS-induced intestinal flora disturbances and lung injury in mice.

Long-term antibiotic treatment can reduce bacterial load and inflammation in the respiratory tract of patients with COPD. However, the side effects of long-term use of antibiotics and bacterial resistance are common (Rusu and Buta, 2021). In the future, we can use the known key lung target bacteria for the treatment of COPD to develop more targeted safe and effective drugs. In general, the feasibility of pulmonary and intestinal microbiota as new targets for early diagnosis and clinical treatment of COPD needs to be further clarified through the active combination of conventional treatment methods and various emerging programs or the feasibility of the future successful application of lung intestinal microbiota as the target of COPD prevention and treatment strategy.

### 4.3 Lung cancer

Lung cancer (LC) is one of the deadliest malignancies with increasing morbidity and mortality worldwide. In the past few decades, despite many breakthroughs in tumor-targeted therapy and immunotherapy, there are still many limitations, such as the emergence of drug resistance, recurrence, and metastasis of lung cancer. Therefore, there is an urgent need to find new ways to treat lung cancer. Approximately 95% of lung cancers are classified as small cell lung cancer (SCLC) or non-small cell lung cancer (NSCLC) (Suster and Mino-Kenudson, 2020). In addition to genetic and environmental factors, the microbiome also plays an important role in the development of lung cancer. It has been reported that the occurrence of surface boundary tumors is usually related to the destruction of the host mucosal immune barrier. When the mucosal surface is damaged, if the damage can be repaired in time, the microenvironment of the original tissue and the symbiotic microbiome will be rebuilt. Otherwise, the damage will continue to intensify and lead to repeated bouts of inflammation that may eventually trigger cancer (Goto, 2022).

Zhang et al. (2018) sequenced the highly variable V1-V2 region of the 16S rRNA gene in stool samples from lung cancer patients and healthy volunteers. The results showed that compared with the healthy control group, the levels of *Bacteroidetes* and *Fusobacteria* in the lung cancer group were higher, but the levels of *Firmicutes* and *Proteobacteria* were significantly decreased. This suggests a potential link between gut bacteria and lung cancer. Zhuang et al. (2019) sequenced the intestinal microbiota 16S rRNA of 30 LC patients and 30 healthy controls and found that in the process of LC, intestinal flora is significantly maladjusted, in which the decreased levels of *Actinobacteria* and *Bifidobacterium* and the increased levels of *Enterococcus* are significantly correlated with lung cancer, and the decreased normal function of intestinal microflora will affect the progression of lung cancer. Liu et al. (2019) analyzed fecal samples from 30 lung cancer patients and 16 healthy controls by 16S rRNA gene amplification sequencing, and the results showed that compared with the healthy control group, the intestinal microbial community in the lung cancer group presented a microbial ecosystem with elimination, low density, and loss of bacterial diversity. It is characterized by an increase in pathogenic bacteria (*Enterobacteri*aceae, *Streptococcus, Prevotell*a, etc.) and a decrease in probiotics (*Blautia, Coprococcus, Bifidobacterium*, and *Lachnospiraceae*). And the abundance of some major metabolism-related pathways in lung cancer is reduced. In summary, *Enterobacteriaceae, Streptococcus, Prevotella, Coprococcus, Bifidobacterium*, and *Lachnospiraceae* have often been reported as the most relevant microbiome for lung cancer. This means that the gut flora can serve as microbial markers and contribute to the derived metabolites, development, and differentiation of the lung cancer system. In addition, Fang et al. (2023) maintained the circadian rhythm synchronization of C57BL/6J lung cancer mice by time-limited feeding. 16S rRNA sequencing and non-targeted metabolome sequencing found that the intestinal flora components of lung cancer mice returned to the level of non-lung cancer mice and slowed down the growth rate of tumors, demonstrating the possibility of regulating fecal flora and thus inhibiting lung cancer.

The intestinal flora of lung cancer patients is different from that of healthy people. More and more studies have gradually confirmed that by regulating intestinal flora, increasing probiotics, and reducing harmful bacteria, tumor inhibition signaling pathway can be more activated, anti-tumor immunity can be enhanced, apoptosis of cancer cells can be induced, or recurrence and metastasis can be prevented, and it can play a synergistic role with tumor therapeutic drugs. It can bring more clinical benefits to patients. However, first, due to technological development and ethical limitations, most studies are currently conducted in laboratory mice, and the sample size of studies in human diseases is also small; second, the role of microbial components such as fungi and viruses in lung cancer, in addition to bacteria, has been largely unexplored, partly due to their relatively low abundance. And there is a lack of well-characterized reference genomes. Emerging advances in organoid technology have made 3D studies of human lung tissue possible. Regarding the feasibility of using lung organoids to approach the mechanisms of cell–cell interactions in lung tissue, future studies should attempt to use organoids to better model and explore the role of microbes in lung cancer and possible molecular mechanisms, as it has successfully studied the microbiota associated with colorectal cancer (Pleguezuelos-Manzano et al., 2020).

### 4.4 Asthma

Asthma is a heterogeneous disease that includes multiple phenotypes and varies in clinical and pathophysiological features (Kaur and Chupp, 2019). The main clinical symptoms were shortness of breath, chest tightness, dizziness, fatigue, etc. (Stern et al., 2020), and the World Health Organization reports that more than 262 million people suffer from asthma and more than 400,000 people die (GBD 2019 Diseases and Injuries Collaborators, 2020). The pathogenesis of asthma is unknown, but the disease is associated with multiple genetic, environmental, infectious, and nutritional factors (Miller et al., 2021). Epidemiological surveys and experimental evidence support that changes in the intestinal immune response can directly lead to the development of pulmonary allergic diseases (Gans and Gavrilova, 2020). The “microbial hypothesis” believes that the abnormal immune response caused by the imbalance of intestinal microbiota caused by external factors (such as changes in diet structure, improvement of sanitary conditions, and use of antibiotics) is the key to the occurrence of airway allergic inflammation factor (Bai et al., 2020; Hakozaki et al., 2020; Ye et al., 2022).

Asthma is the most prevalent childhood disease in Western countries (Mahesh and Ramamurthy, 2022). Several studies have linked dysregulation of the gut microbiome early in life to an increased risk of asthma later in life. Compared with non-asthmatic children, school-age children with asthma showed lower gut microbiome diversity before 1 month of age (Abrahamsson et al., 2014). Colonization of *Clostridium difficile* species (phylum *Firmicutes*) at 1 month of age is associated with wheezing and asthma at 6–7 years of age (Van Nimwegen et al., 2011). Another study analyzed the gut microbiome of infants at risk for asthma in the first 100 days of life and discovered that the relative abundance of the genera *Lachnospira, Veillonella, Faecalibacterium* (phylum *Firmicutes*), and *Rothia* (phylum *Actinobacteria*) was significantly decreased in these children (Arrieta et al., 2015). The same group of authors confirmed bacterial dysbiosis of the gut microbiota in another study and showed that opposite changes in the relative abundance of *Lachnospira* and *Clostridium neonatale* in newborns within 3 months of birth are associated with the development of preschool asthma (Stiemsma et al., 2016). In a recent metabolomics-based approach, stool samples from children with asthma aged 4–7 years were compared with healthy children, focusing on gut metabolites such as amino acids or butyrate (Chiu et al., 2019). The results of taxonomic classification showed that among gut bacteria, the phyla *Firmicutes* (67.8%), *Actinobacteria* (20.7%), and *Bacteriodetes* (8.4%) accounted for 97% of all the sequences analyzed. Compared with healthy controls, children with asthma had significantly lower abundance of genera *Faecalibacterium* and *Roseburia* (phylum *Firmicutes*), while increased abundance in the genera *Enterococcus* and *Clostridium* (phylum *Firmicutes*). *Firmicutes* bacteria are dysbiosis bacteria that are significantly less common in children with asthma and may therefore be associated with increased asthma risk.

The intestinal microbiota is closely related to the pathogenesis of asthma (Barcik et al., 2020). Intestinal microbiota can regulate the immune function of the body by increasing the level of SCFAs, and then affect the occurrence and development of asthma (Tagé et al., 2024). However, due to the close and complex relationship between intestinal microbiota and asthma, the specific regulation mechanism of intestinal microbiota participating in immune tolerance is not completely clear, so it needs to be further elucidated (Tan et al., 2023).

### 4.5 Pulmonary arterial hypertension

Pulmonary arterial hypertension (PAH) is a malignant pulmonary vascular disease characterized by increased pulmonary vascular resistance, pulmonary vasoconstriction, and right ventricular hypertrophy, which eventually leads to right heart failure and death (Luna-López et al., 2022). The main pathophysiological mechanisms of PAH are endothelial injury, pulmonary vascular remodeling, and *in situ* thrombosis (Mandras et al., 2020). Due to the lack of screening methods and biomarkers for early detection, patients with PAH often present with severe right ventricular dysfunction at the time of diagnosis (Wedgwood et al., 2020). The 1-, 3-, and 5-year survival rates of PAH patients are 90.4%, 76.2%, and 65.4%, respectively, thus increasing the socioeconomic burden in China and the West (Farber et al., 2015), and no new therapeutic approach for PAH has been found yet. Advances in genomics and metabolomics have gradually revealed the role of gut microbiota (GM) and its metabolites in cardiovascular diseases.

In an experimental PAH rat model, the researchers systematically analyzed the gut microbiome composition of stool samples from rats with pulmonary arterial hypertension (PAH) and found that the gut microbiota was significantly dysregulated during PAH, with a three-fold increase in the ratio of Firmicutes to Bacteroides (Callejo et al., 2018). Hong et al. (2021) conducted 16S rRNA gene sequencing analysis on stool samples of PAH rats, the results showed that compared with the control group, for the phylum level, the relative abundance of Firmicutes, Proteobacteria, and Actinobacteria increased while the relative abundance of *Bacteroidota* and *Spirochaetota* decreased. For the class level, *Bacilli* decreased in relative abundance and *Firmicutes–Clostridia* increased in relative abundance. For the genus level, the relative abundance of *Allobaculum, Ralstonia*, and *Bifidobacterium* increased, while the relative abundance of *Lactobacillus* and *Romboutsia* decreased. Similarly, intestinal microbiota imbalances have been found in patients with pulmonary hypertension. Kim et al. (2020) compared the fecal microbiome of 18 patients with type 1 PAH and 13 healthy controls by shotgun metagenomics and found that there was fewer beneficial short-chain fatty acid (SCFA) producing bacteria in the stool of PAH patients, such as *Coprococcus, Butyrivibrio, Lachnospiraceae, Eubacterium*, and *Clostridia*. These changes in PAH biogroups may disrupt metabolic and immune homeostasis, and influence the pulmonary vasculature. In conclusion, the dysbiosis of gut microbiota in PAH animals and patients highlights the role of gut microbiota in PAH and provides new targets for PAH diagnosis and treatment.

In summary, we can conclude that microbial communities interact with PAH because different characteristics of gut microbiota were found after exposure to factors such as hypoxia and MCT, and PAH models were successfully established. At the same time, long-term PAH can lead to intestinal venous congestion and then lead to intestinal dysbiosis. Microbiota-based therapy may become a new treatment strategy for PAH, but relevant clinical evidence is lacking. Large-scale clinical studies need to be designed to validate the efficacy of microbiota-based PAH therapies. For example, whether patients undergoing colectomy are at risk for altered PAH and whether patients with PAH treated with FMT or probiotics have improved signs and symptoms of PAH.

### 4.6 COVID-19

Severe acute respiratory syndrome coronavirus 2 (SARS-CoV-2, formerly known as 2019-nCoV) is an enveloped, single-stranded genomic RNA virus (+ssRNA) that is the cause of coronavirus disease 2019 (COVID-19). COVID-19 infection was first reported in Wuhan, China, in December 2019 and has since spread rapidly around the world (Gupta et al., 2020; Wang et al., 2020; de Oliveira et al., 2021). Previous studies have shown that gut microbiota involved in the development and function of the innate and adaptive immune systems may play an important role in the pathogenesis of COVID-19 (Zheng et al., 2020). Depletion of SCFA-producing microbiota in COVID-19 or severe disease has also been associated with cytokine alterations (Lv et al., 2021; Yeoh et al., 2021; Zhang et al., 2022). Therefore, a comprehensive investigation of the interrelationships among gut microbes, metabolites, and cytokines, as they influence COVID-19, should expand our knowledge of disease pathogenesis.

A recent clinical study of COVID-19 patients showed that a bacterial formula administrated orally: *Streptococcus thermophilus* DSM 32345, *L.acidophilus* DSM 32241, *L. helveticus* DSM 32242, *L. paracasei* DSM 32243, *L. plantarum* DSM 32244, *L. brevis* DSM 27961, *B. lactis* DSM 32246, *B. lactis* DSM 32247 (SivoBiome^®^ in USA) reduces the risk of respiratory failure and accelerates the resolution of diarrhea (d'Ettorre et al., 2020). One study assessed the presence of SARS-CoV-2 in throat swabs and stool samples throughout infection. Stool and respiratory swabs were obtained every 1–2 days until two consecutive tests were negative. The results showed that stool samples remained positive for ~5 weeks after respiratory samples tested negative for viral RNA in this patient cohort (Wu et al., 2020). This, together with the fact that some patients with this condition have diarrhea, points to the distinct possibility of involvement of the gut–lung axis and, possibly, the gut microbiome (Chan et al., 2020). Thus, the presence or persistence of the virus in the oropharynx and stool highlights that SARS-CoV-2 is not restricted to the lungs and suggests the possibility of fecal–oral transmission. In an observational study, Fan et al. (2020) evaluated the lung microbiota of 20 deaths from COVID-19. Members of *Acinetobacter, Brevundimonas, Burkholderia, Chryseobacterium, Sphingosine*, and *Enterobacteriaceae* dominate the lung microbiota in these patients. *Enterobacteriaceae*, which includes species commonly found in the gut microbiome, including some pathogenic microorganisms such as *Enterobacter, Escherichia coli, Klebsiella*, and *Proteus*, were detected in the lungs of deceased COVID-19 patients. In the genus Acinetobacter, *A baumannii* is associated with multidrug-resistant infections and mortality (Doi et al., 2015). In addition, Kolhe et al. (2021) compared the nasopharyngeal microbiota characteristics of elderly COVID-19 patients with healthy individuals and found that patients were enriched in Cyanobacterial taxa at the phylum level, and the number of *Litoricola, Amylibacter, Balneola*, and *Aeromonas* at the genus level was increased trends, suggesting that nasal microbiota is associated with SARS-CoV-2 severity in elderly patients.

Shotgun metagenomic sequencing and metabolomics were performed on stool samples from 112 hospitalized patients with SARS-CoV-2 infection and 112 non-COVID-19 controls matched for important confounders, as well as plasma cytokine measurements. Multiple associations were identified between COVID-19-associated microbes (e.g., oral microbes and producers of short-chain fatty acids) and gut metabolites (e.g., branched-chain and aromatic amino acids, short-chain fatty acids, carbohydrates, neurotransmitters, and vitamin B6). Both are also associated with inflammatory cytokine dynamics (e.g., interferon-γ, interferon-λ3, interleukin-6, CXCL-9, and CXCL-10) (Nagata et al., 2023). It is therefore reasonable to hypothesize that adjuvant therapies based on gut–lung axis modulation and restoration of true life may be an important therapeutic approach to limit the harmful consequences of COVID-19.

### 4.7 Cystic fibrosis

Cystic fibrosis (CF) is caused by mutations in the cystic fibrosis transmembrane conductance regulator (CFTR) gene, which results in altered chloride and bicarbonate secretion and the accumulation of abnormally thick mucus in the lungs and intestine, affecting more than 70,000 people worldwide (Lopes-Pacheco, 2020). Recent microbiological studies have identified the gut microbiota as an important player in gut and lung health outcomes in CF patients (Testa et al., 2022). Common gastrointestinal symptoms in CF patients include abdominal pain, bloating, bloating, steatosis, slow weight gain, and constipation (Meeker et al., 2020). CFTR is expressed in intestinal epithelial cells, and its dysfunction in CF leads to the deficiency of CFTR protein, resulting in various physiological and biochemical imbalances. These symptoms include mucus thickening and oozing due to chloride channel dysfunction, defective bicarbonate secretion altering the intestinal pH environment, prolonged intestinal transport time, pancreatic insufficiency, increased intestinal inflammation, and altered immune mechanisms with impaired intestinal barrier function (Vernocchi et al., 2018; Wang et al., 2019; Kristensen et al., 2020).

Changes in a particular taxa are usually represented by an expansion of known pathogens and a relative reduction in genera with beneficial members. Along with reduced gut microbial diversity, there is a significant alteration of microbial composition in patients with CF, for example, an increase in *Firmicutes*, a reduction in *Bacteroidetes*, along with a higher abundance of pro-inflammatory microbiota such as those belonging to *Enterobacteriaceae, Streptococcus*, and *Veillonella* are consistently reported in patients with CF (Hayden et al., 2020; Kristensen et al., 2020). Stool samples from children with CF were examined using 16S rRNA sequencing and metabolomics. Vernocchi et al. (2018) demonstrated abundant *Propionibacterium, Staphylococcus* and *Clostridiaceae* along with reduced abundance of *Eggerthella, Eubacterium, Ruminococcus, Dorea, Faecalibacterium prausnitzii (F. prausnitzii*), and *Lachnospiraceae* and further noted that the resulting dysbiosis was associated with increased expression of metabolites such as gamma-aminobutyric acid (GABA), choline, ethanol, propylbutyrate, and pyridine, and reduced levels of sarcosine, 4-methylphenol, uracil, glucose, acetate, phenol, benzaldehyde, and methyl acetate.

The quantity, diversity, and dominant microbiota of intestinal microbiota in CF patients may be used as biomarkers and a way to evaluate prognosis. Although CF is monogenic, it is a multifactorial disease, and both genotype and microbiome profile are key interrelated factors in disease progression. Thus, microbiome–genome interactions are important. Metagenomics, now applied essentially in a gene-targeting fashion, has been able to characterize bacterial communities and, to a lesser extent, fungi in the CF lung niche in great detail. With the help of bioinformatics advances, the integration of genetic and microbiological data to study the interactions between microbiota and their metabolites may lead to the establishment of individual microbial signatures of CF patients to understand the unique disease progression of each patient and achieve precise individual assessment and treatment.

## 5 Therapeutic strategies for respiratory diseases-microbiome modulation

### 5.1 Probiotics for respiratory diseases

Probiotics are defined as live microorganisms that, if ingested in sufficient quantities, may have a beneficial effect on the health of the host. In contrast to other techniques such as FMT, probiotic therapy provides targeted modulation of the gut microbiota through the addition of “healthy” probiotics (Kim et al., 2019). *Clostridium butyricum*, as a symbiotic bacteria in the human gut, can promote the production of short-chain fatty acids such as acetic acid, propionic acid, and butyric acid, as well as vitamins such as B vitamins, folic acid, and vitamin K in the intestinal metabolism process, and these nutrients further play a positive role in the body (Cao et al., 2022). Many studies have shown that *C. butyricum* has certain preventive and therapeutic effects on bronchial asthma, lung cancer, lung infection, and other diseases and is a probiotic with great development potential. For example, Tian et al. (2019) and Cong et al. (2022) found that oral administration of *C. butyricum* could reduce the incidence of adverse events during chemotherapy in lung cancer patients, enhance the innate immune response of the body, reduce the level of inflammation, regulate the abundance of beneficial bacteria and pathogenic bacteria in the intestine differently, and help maintain the homeostatic state of intestinal flora. It can be an important adjunct drug in the treatment of lung cancer. Animal experiments have also shown that *C. butyricum* CGMCC0313-1 can reduce ovalbumin-induced allergic airway inflammation in mice (Juan et al., 2017; Li L. et al., 2022). In a mouse model of allergic asthma, oral administration of Clostridium butyrate can inhibit airway remodeling and airway hyperreactivity, reduce airway inflammation, and reduce mast cell degranulation. Overactivation of Th2 cells and underactivation of Th1 lead to Th1/Th2 balance disturbance, which plays an important role in the progression of allergic asthma. *C. butyricum* can reverse the Th1/Th2 imbalance in the airway of asthmatic mice and increase the expression of the anti-inflammatory cytokine IL-10 (Juan et al., 2017). Further studies showed that *C. butyricum* can not only restore Th1/Th2 balance in mice with allergic asthma but also improve lung autophagy and inhibit the NF-κB/NLRP3 inflammatory signaling pathway in the lungs, and further relieve allergic airway inflammation (Li L. et al., 2022).

In recent years, the use of probiotics has been increasing rapidly, and the safety of probiotics is the most important issue (Ahire et al., 2023). Because different probiotic strains may have different safety profiles, care must be taken when using probiotics in patients with severe immunodeficiency, malnutrition or cancer (Kan et al., 2024). The glossary is shown in Table 1.

**Table 1:** Glossary.

**Term**	**Consensus definition**
Microbiota	16S rRNA surveys are used to taxonomically identify the microorganisms in the environment
Metagenomics	The genes and genomes of the microbiota, including plasmids, highlight the genetic potential of the population
Microbiome	The genes and genomes of the microbiota, as well as the products of the microbiota and the host environment
Prebiotics	Gut microbiota-accessible dietary fibers that nourish and promote the growth of beneficial bacteria
Probiotics	Beneficial live microorganisms found in fermented foods and supplements
Microdysbiosis	The disturbance of the ecosystem is caused by the destruction of the microecological balance

### 5.2 Dietary fiber supplement

Dietary fiber refers to the sum of polysaccharides and a small amount of lignin that come from plants and are not hydrolyzed by digestive enzymes in the small intestine, mainly cellulose, hemicellulose, and pectin (Asp, 1987). Dietary fiber intake has a significant impact on the diversity and abundance of microflora in the intestinal microbiome (Cronin et al., 2021). Some animal experiments have also shown that dietary fiber can maintain the integrity of intestinal mucosal structure, protect its barrier function, and prevent bacterial and endotoxin translocation (Desai et al., 2016; Paudel et al., 2024). Studies have found that an unhealthy Western-style diet is associated with an increased risk of COPD and an accelerated decline in lung function (van Iersel et al., 2022), while dietary fiber intake, through anti-inflammatory (King et al., 2003; Ma et al., 2006, 2008) as well as indirect antioxidant properties (Saura-Calixto, 2011), may protect lungs against inflammation and prevent COPD, especially through modulation of the innate immune system via the gut–liver–lung axis (Young et al., 2016) and enhancing the bioavailability of antioxidants (Palafox-Carlos et al., 2011). In addition, studies have found that a higher intake of dietary fiber and yogurt in the daily diet may help reduce lung cancer risk, possibly because dietary fiber-rich foods contain prebiotics, while yogurt is a common source of probiotics, both of which can improve and regulate the composition and function of gut microbiota (Yang et al., 2020). Multiple studies have confirmed the protective effect of dietary fiber on allergic asthma. A study in model mice with asthma showed that eosinophilic airway inflammation, IgE levels, and Th2-related inflammatory mediators were reduced after treatment with dietary fiber, while airway obstruction and lung function were improved (Manni et al., 2021). Another study noted that in adults with severe asthma, dietary fiber intake was inversely associated with airway eosinophilia and decreased lung function (Berthon et al., 2013). Dietary fiber intake in adults can also regulate inflammatory responses, reduce the risk of asthma in the population, and prevent coughing, wheezing, and sputum production (Saeed et al., 2020).

In addition, the researchers pointed out that based on fresh foods rich in vitamin C, dietary fiber, and dietary polyphenols, such as fruits, vegetables, and whole grains, increasing the intake of probiotics and prebiotics plays an important role in respiratory virus infection (Yue et al., 2022). Trompette et al. (2018) found that the overall abundance of intestinal microflora in influenza-infected mice was reduced, the structure of intestinal microflora could be significantly improved after feeding fermentable dietary fiber, and the relative abundance of beneficial bacteria such as *Bifidobacterium* and *Bacteroides* could be increased, thus generating SCFAs (especially butyric acid) and promoting the extinction of influenza infection. Antunes et al. (2019) observed the protective effect of a high-fiber diet (citrus cellulose and pectin) on lung tissue of mice infected with respiratory syncytial virus and found that mice with a high-fiber diet had lower morbidity, less weight loss, and a lot of improvement in lung tissue inflammation and pathological damage. High-fiber-diet-fed mice had an increase of intestinal bacterial components of *Lachnospiraceae* that produce SCFAs. SCFAs reach lung tissue through systemic circulation, activate G-protein-coupled receptor 43(GPR43) of the alveolar epithelium, release IFN-β, and thus exert antiviral and lung protective effects. In conclusion, dietary fiber intake is particularly important for the dietary management of pulmonary diseases and should be regarded as the key dietary guidance content in the management model of related diseases.

### 5.3 Short-chain fatty acid supplementation regulates intestinal homeostasis

Short-chain fatty acids (SCFAs), as microbial metabolites, are essential for maintaining a healthy gut and overall health. They are produced by fermenting dietary fiber with beneficial gut bacteria and play a vital role in energy metabolism, intestinal barrier integrity, immune function, and overall inflammation regulation. SCFA metabolism disorders are also closely related to the occurrence of allergic inflammation, asthma, and other respiratory diseases (Verma et al., 2024). Butyric acid, as one of the main members of short-chain fatty acids, has been shown to play an important role in different aspects of lung diseases such as allergic asthma, chronic obstructive pulmonary disease, and pulmonary fibrosis (Kabel et al., 2016; Yip et al., 2021). Butyrate is mainly produced by the digestion of dietary fiber in the intestine, can supply energy to the body through the oxidation of fatty acids in the body, and is the main energy supplier for intestinal epithelial cells (Liu et al., 2017). Studies have found that the epithelial barrier of colon mucosa relies on the oxidation of butyric acid to maintain, and the insufficient content of butyric acid in the intestine or the increase of sulfide in the intestine inhibits the action of the REDOX system on butyric acid, which will lead to inflammation (Roediger, 1980). However, as a ligand of GPCR, butyrate is an agonist of GPR41, GPR43, and GPR109A (Li G. et al., 2021), which can induce the differentiation of Treg cells and T cells by activating the GPCR signal of intestinal epithelial cells and promote the anti-inflammatory properties of colon macrophages and dendritic cells (Singh et al., 2014).

Although most butyrate is absorbed and consumed in the colon, the literature suggests that several roles of this metabolite in different peripheral tissues are significantly associated with lung disease (Blaak et al., 2020). The systemic effects of butyrate depend on its uptake by intestinal epithelial cells (IEC) and the subsequent distribution of this metabolite in the bloodstream (Salvi and Cowles, 2021). The study found that butyrate presented a potent antifibrotic effect by inhibiting mitochondrial elongation in TGF-β induced pulmonary fibrosis, increasing their mitochondrial membrane potential and ATP, NADH, and NADH/NAD ratio, affecting myofibroblast differentiation (Park et al., 2021). Butyrate attenuates BLM-induced lung fibrosis in rats. Animals receiving BLM in combination with butyrate presented a reduction in body weight loss and an improvement in the levels of inflammatory mediators and immune cells in their bronchoalveolar lavage compared to those receiving BLM alone, demonstrating a possible prophylactic role of this SCFA to certain conditions of pulmonary fibrosis (Williams et al., 2019). *In vitro*, cigarette smoke extract was shown to induce human embryonic lung fibroblasts to differentiate into myofibroblasts by causing endoplasmic reticulum stress—a condition that is associated with fibrosis and could be suppressed to some extent by the treatment with 4-phenyl butyric acid (4-PBA)—a butyrate analog compound (Gasse et al., 2009). In addition, butyrate was shown to inhibit the proliferation and migration of A549 human lung cancer epithelial cells *in vitro* by up-regulating miR-3935 expression (Xiao et al., 2018). Therefore, the supplementation of short-chain fatty acids, including butyric acid, may provide new ideas for the prevention and treatment of respiratory diseases.

## 6 Conclusions

With the study of the lung–gut theory in recent years, the relevant biological connection between the lung and the large intestine has been further confirmed, providing convincing evidence for the “lung–gut combined therapy.” The “gut–lung axis” is essentially a two-way communication and action hub between the gut microbiota and the lungs, when external factors (such as diseases, drugs, etc.) change and regulate the immune response. There are two main ways in which the microbiota affects the function of the respiratory system. On the one hand, some metabolites of intestinal microorganisms, such as short-chain fatty acids (SCFAs) and deaminotyrosine (DAT) enter the lung tissue through the blood and participate in lung immune function. Whereas. on the other hand, intestinal immune cells and immune factors enter the respiratory system through blood circulation and participate in the inflammatory process. These findings may provide important assistance to the research on the pathogenesis and influencing factors of respiratory diseases in the future, and provide new strategies for the diagnosis and treatment of diseases (Table 2).

**Table 2:** Changes in intestinal flora in respiratory diseases.

**The type of respiratory disease**	**Patient/ animal model**	**The changes in gut microbiome**	**Results**	**References**
Idiopathic pulmonary fibrosis	Patients	*Firmicutes, Actinobacteria, Devosia, Clostridiales, Alloprevotella, and Rikenellaceae_RC9↓*	The intestinal microbiota of patients with pulmonary fibrosis had significant changes	Zhou et al., 2019
	Animal model	*Helicobacter* ↓ *Dubosiella* ↑	The abundance of *Dubosiella* was significantly positively correlated with the level of betaine in serum	Gong et al., 2021
Chronic obstructive pulmonary disease	Patients	*Bacteroides, Roseburia*, and *Lachnospira ↓* *Streptococcus, Rothia, Romboutsia, Intestinibacter*, and *Escherichia ↑*	COPD patients have an imbalance in their gut flora	Bowerman et al., 2020
	Patients	*Ruminococcaceae* and *Lachnoclostridium ↓* *Aerococcus* and *Fusobacterium↑*		Chiu et al., 2021
	Animal model	*Escherichia-Shigella ↑*	The onset and development of COPD were associated with the imbalance of 41 differential metabolites in plasma, bronchoalveolar lavage fluid, and feces, and 82 bacteria at the levels of phylum, class, order, family, and genus from lung and intestine	Jiao et al., 2022
Lung cancer	Patients	*Firmicutes* and *Proteobacteria↓* *Bacteroidetes* and *Fusobacteria↑*	This suggests a potential link between gut bacteria and lung cancer	Zhang et al., 2018
	Patients	*Actinobacteria* and *Bifidobacterium↓* *Enterococcus↑*	The decreased normal function of intestinal microflora will affect the progression of lung cancer	Zhuang et al., 2019
	Patients	*Blautia, Coprococcus, Bifidobacterium* and *Lachnospiraceae↓* *Enterobacteriaceae, Streptococcus, Prevotella ↑*	The intestinal microbial community in the lung cancer group presented a microbial ecosystem with elimination, low density, and loss of bacterial diversity	Liu et al., 2019
Asthma	Patients	The genera *Lachnospira, Veillonella, Faecalibacterium* (phylum *Firmicutes*), and *Rothia* (phylum *Actinobacteria*) ↓	Dysregulation of the gut microbiota early on has been linked to an increased risk of asthma later in life	Arrieta et al., 2015
	Patients	Genera *Faecalibacterium* and *Roseburia* (phylum *Firmicutes*)↓ Genera *Enterococcus* and *Clostridium* (phylum *Firmicutes*)↑	*Firmicutes* bacteria are dysbiosis bacteria that are significantly less common in children with asthma and may therefore be associated with increased asthma risk	Chiu et al., 2019
Pulmonary arterial hypertension	Animal model	*Bacteroidota* and *Spirochaetota ↓* *Firmicutes, Proteobacteria*, and *Actinobacteria ↑* *Bacilli ↓* *Firmicutes-Clostridia ↑* *Lactobacillus* and *Romboutsia ↓* *Allobaculum, Ralstonia*, and *Bifidobacterium ↑*	Pulmonary hypertension causes an imbalance in the gut flora	Hong et al., 2021
	Patients	*Coprococcus, Butyrivibrio, Lachnospiraceae, Eubacterium*, and *Clostridia ↓*	These changes in PAH biogroups may disrupt metabolic and immune homeostasis, and influence the pulmonary vasculature	Kim et al., 2020
COVID-19	Patients	*Acinetobacter, Brevundimonas, Burkholderia, Chryseobacterium, Sphingosine*, and *Enterobacteriaceae* ↑	The virus is not confined to the lungs and suggests the possibility of fecal–oral transmission	Fan et al., 2020
Cystic fibrosis	Patients	*Bacteroidetes ↓* *Firmicutes, Enterobacteriaceae, Streptococcus*, and *Veillonella ↑*	Along with reduced gut microbial diversity, there is a significant alteration of microbial composition in patients with CF	Hayden et al., 2020; Kristensen et al., 2020
	Patients	*Eggerthella, Eubacterium, Ruminococcus, Dorea, Faecalibacterium prausnitzii (F. prausnitzii)*, and *Lachnospiraceae ↓* *Propionibacterium, Staphylococcus*, and *Clostridiaceae* ↑	CF can lead to microecological dysregulation and lower metabolite content	Vernocchi et al., 2018

While previous studies have elaborated the overall profile of the lung microbiota, suggesting important correlations between the microbiota and immune response homeostasis, limitations of the current study remain. (a) The relatively low abundance of the microbiota in the lung poses challenges to the isolation, culture, and identification of microorganisms; (b) mouse models have significant limitations in preclinical studies. One study showed that 85% of the bacterial genera found in the mouse gut microbiome have not been found in humans (Hugenholtz and de Vos, 2018); (c) the microbiota in the three individuals was often translocated due to changes in the host diet or lifestyle and competitive exclusion between species; (d) the research on syndrome differentiation and treatment of lung diseases from the perspective of “combined treatment of lung and intestine” is not deep enough, and the specific mechanism of “combined treatment of lung and intestine” is still not completely clear.

In summary, the academic community has made significant progress in the field of research on the regulation of gut microbiota on lung immunity, elucidating the key role of the microbiota in the lung in establishing local immune balance and defending against external pathogens. With the increasing understanding of microbiology and immunology, the modulation of gut microbiota is a potential target for the treatment of lung diseases, and we can identify potential inflammatory markers and microorganisms through better animal models and high-quality clinical study targets to explore new mechanisms and treatments for diseases. In addition, studies should control for baseline differences in individual microbiomes, including sex, age, race, comorbidities, drug use (including antibiotics and probiotics), diet and lifestyle, and environment, provide a more standardized measurement method for microbiome research, verify clinical data with a larger sample size, and further clarify the regulation mechanism of intestinal microbiome on the treatment of respiratory diseases, to apply it to clinical precision treatment and better achieving therapeutic effect.

## Author contributions

MS: Writing – original draft. FL: Writing – review & editing. DY: Writing – review & editing. YW: Writing – review & editing. PC: Writing – review & editing. SL: Funding acquisition, Supervision, Writing – review & editing.

## References

[B1] Abrahamsson T. R. Jakobsson H. E. Andersson A. F. Björkstén B. Engstrand L. Jenmalm M. C. (2014). Low gut microbiota diversity in early infancy precedes asthma at school age. Clin. Exp. Allergy 44, 842–850. 10.1111/cea.12253 24330256

[B2] Ahire J. J. Rohilla A. Kumar V. Tiwari A. (2023). Quality management of probiotics: ensuring safety and maximizing health benefits. Curr. Microbiol. 81:1. 10.1007/s00284-023-03526-3 37935938

[B3] Aishwarya S. Gunasekaran K. Anita Margret A. (2022). Intermodulation of gut-lung axis microbiome and the implications of biotics to combat COVID-19. J. Biomol. Struct. Dyn. 40, 14262–14278. 10.1080/07391102.2021.1994875 34699326

[B4] Allard B. Panariti A. Martin J. G. (2018). Alveolar macrophages in the resolution of inflammation, tissue repair, and tolerance to infection. Front. Immunol. 9:1777. 10.3389/fimmu.2018.01777 30108592 PMC6079255

[B5] Antunes K. H. Fachi J. L. de Paula R. da Silva E. F. Pral L. P. Dos Santos A. Á.. (2019). Microbiota-derived acetate protects against respiratory syncytial virus infection through a GPR43-type 1 interferon response. Nat. Commun. 10:3273. 10.1038/s41467-019-11152-6 31332169 PMC6646332

[B6] Arrieta M. C. Stiemsma L. T. Dimitriu P. A. Thorson L. Russell S. Yurist-Doutsch S.. (2015). Early infancy microbial and metabolic alterations affect risk of childhood asthma. Sci. Transl. Med. 7:307ra152. 10.1126/scitranslmed.aab2271 26424567

[B7] Asp N. G. (1987). Dietary fibre–definition, chemistry and analytical determination. Mol. Aspects Med. 9, 17–29. 10.1016/0098-2997(87)90014-8 3031413

[B8] Bai C. Liu T. Xu J. Ma X. Huang L. Liu S.. (2020). Effect of high calorie diet on intestinal flora in LPS-induced pneumonia rats. Sci. Rep. 10:1701. 10.1038/s41598-020-58632-0 32015367 PMC6997398

[B9] Bai X. Wei H. Liu W. Coker O. O. Gou H. Liu C.. (2022). Cigarette smoke promotes colorectal cancer through modulation of gut microbiota and related metabolites. Gut 71, 2439–2450. 10.1136/gutjnl-2021-325021 35387878 PMC9664112

[B10] Barcik W. Boutin R. C. T. Sokolowska M. Finlay B. B. (2020). The role of lung and gut microbiota in the pathology of asthma. Immunity 52, 241–255. 10.1016/j.immuni.2020.01.007 32075727 PMC7128389

[B11] Berthon B. S. Macdonald-Wicks L. K. Gibson P. G. Wood L. G. (2013). Investigation of the association between dietary intake, disease severity and airway inflammation in asthma. Respirology 18, 447–454. 10.1111/resp.12015 23145908

[B12] Blaak E. E. Canfora E. E. Theis S. Frost G. Groen A. K. Mithieux G.. (2020). Short chain fatty acids in human gut and metabolic health. Benef. Microbes 11, 411–455. 10.3920/BM2020.0057 32865024

[B13] Bowerman K. L. Rehman S. F. Vaughan A. Lachner N. Budden K. F. Kim R. Y.. (2020). Disease-associated gut microbiome and metabolome changes in patients with chronic obstructive pulmonary disease. Nat. Commun. 11:5886. 10.1038/s41467-020-19701-0 33208745 PMC7676259

[B14] Bradley C. P. Teng F. Felix K. M. Sano T. Naskar D. Block K. E.. (2017). Segmented filamentous bacteria provoke lung autoimmunity by inducing gut-lung axis Th17 cells expressing dual TCRs. Cell Host Microbe 22, 697–704.e4. 10.1016/j.chom.2017.10.007 29120746 PMC5749641

[B15] Callejo M. Mondejar-Parreño G. Barreira B. Izquierdo-Garcia J. L. Morales-Cano D. Esquivel-Ruiz S.. (2018). Pulmonary arterial hypertension affects the rat gut microbiome. Sci. Rep. 8:9681. 10.1038/s41598-018-27682-w 29946072 PMC6018770

[B16] Cao W. Zheng C. Xu X. Jin R. Huang F. Shi M.. (2022). *Clostridium butyricum* potentially improves inflammation and immunity through alteration of the microbiota and metabolism of gastric cancer patients after gastrectomy. Front. Immunol. 13:1076245. 10.3389/fimmu.2022.1076245 36466862 PMC9714544

[B17] Chan J. F.-W. Yuan S. Kok K.-H. To K. K.-W. Chu H. Yang J.. (2020). A familial cluster of pneumonia associated with the 2019 novel coronavirus indicating person-to-person transmission: a study of a family cluster. Lancet 395, 514–523. 10.1016/S0140-6736(20)30154-9 31986261 PMC7159286

[B18] Chang P. V. Hao L. Offermanns S. Medzhitov R. (2014). The microbial metabolite butyrate regulates intestinal macrophage function via histone deacetylase inhibition. Proc. Natl. Acad. Sci. USA. 111, 2247–2252. 10.1073/pnas.1322269111 24390544 PMC3926023

[B19] Chidambaram S. B. Essa M. M. Rathipriya A. G. Bishir M. Ray B. Mahalakshmi A. M.. (2022). Gut dysbiosis, defective autophagy and altered immune responses in neurodegenerative diseases: tales of a vicious cycle. Pharmacol. Ther. 231:107988. 10.1016/j.pharmthera.2021.107988 34536490

[B20] Chiu C. Cheng M. Chiang M. Kuo Y. Tsai M. Chiu C.. (2019). Gut microbial-derived butyrate is inversely associated with IgE responses to allergens in childhood asthma. Pediatr. Allergy Immunol. 30, 689–697. 10.1111/pai.13096 31206804

[B21] Chiu Y.-C. Lee S.-W. Liu C.-W. Lin R. C.-J. Huang Y.-C. Lan T.-Y.. (2021). Comprehensive profiling of the gut microbiota in patients with chronic obstructive pulmonary disease of varying severity. PLoS ONE 16:e0249944. 10.1371/journal.pone.0249944 33836012 PMC8034725

[B22] Chow E. J. Uyeki T. M. Chu H. Y. (2023). The effects of the COVID-19 pandemic on community respiratory virus activity. Nat. Rev. Microbiol. 21, 195–210. 10.1038/s41579-022-00807-9 36253478 PMC9574826

[B23] Cong J. Zhang C. Zhou S. Zhu J. Liang C. (2022). A pilot study: favorable effects of *Clostridium butyricum* on intestinal microbiota for adjuvant therapy of lung cancer. Cancers 14:3599. 10.3390/cancers14153599 35892858 PMC9332558

[B24] Corrêa-Oliveira R. Fachi J. L. Vieira A. Sato F. T. Vinolo M. A. R. (2016). Regulation of immune cell function by short-chain fatty acids. Clin. Transl. Immunol. 5:e73. 10.1038/cti.2016.17 27195116 PMC4855267

[B25] Cronin P. Joyce S. A. O'Toole P. W. O'Connor E. M. (2021). Dietary fibre modulates the gut microbiota. Nutrients 13:1655. 10.3390/nu13051655 34068353 PMC8153313

[B26] Dang A. T. Marsland B. J. (2019). Microbes, metabolites, and the gut-lung axis. Mucosal Immunol. 12, 843–850. 10.1038/s41385-019-0160-6 30976087

[B27] De Nuccio F. Piscitelli P. Toraldo D. M. (2022). Gut–lung microbiota interactions in chronic obstructive pulmonary disease (COPD): potential mechanisms driving progression to copd and epidemiological data. Lung 200, 773–781. 10.1007/s00408-022-00581-8 36241745

[B28] de Oliveira G. L. V. Oliveira C. N. S. Pinzan C. F. de Salis L. V. V. Cardoso C. R. B. (2021). Microbiota modulation of the gut-lung axis in COVID-19. Front. Immunol. 12:635471. 10.3389/fimmu.2021.635471 33717181 PMC7945592

[B29] de Vos W. M. Tilg H. Van Hul M. Cani P. D. (2022). Gut microbiome and health: mechanistic insights. Gut 71, 1020–1032. 10.1136/gutjnl-2021-326789 35105664 PMC8995832

[B30] Desai M. S. Seekatz A. M. Koropatkin N. M. Kamada N. Hickey C. A. Wolter M.. (2016). A dietary fiber-deprived gut microbiota degrades the colonic mucus barrier and enhances pathogen susceptibility. Cell 167, 1339–1353.e21. 10.1016/j.cell.2016.10.043 27863247 PMC5131798

[B31] d'Ettorre G. Ceccarelli G. Marazzato M. Campagna G. Pinacchio C. Alessandri F.. (2020). Challenges in the management of SARS-CoV2 infection: the role of oral bacteriotherapy as complementary therapeutic strategy to avoid the progression of COVID-19. Front. Med. 7:389. 10.3389/fmed.2020.00389 32733907 PMC7358304

[B32] Dickson R. P. Erb-Downward J. R. Martinez F. J. Huffnagle G. B. (2016). The microbiome and the respiratory tract. Annu. Rev. Physiol. 78, 481–504. 10.1146/annurev-physiol-021115-105238 26527186 PMC4751994

[B33] Doi Y. Murray G. Peleg A. (2015). Acinetobacter baumannii: evolution of antimicrobial resistance—treatment options. Semin. Respir. Crit. Care Med. 36, 085–098. 10.1055/s-0034-1398388 25643273 PMC4465586

[B34] Dumas A. Bernard L. Poquet Y. Lugo-Villarino G. Neyrolles O. (2018). The role of the lung microbiota and the gut-lung axis in respiratory infectious diseases. Cell. Microbiol. 20:e12966. 10.1111/cmi.12966 30329198

[B35] Ekbom A. Brandt L. Granath F. Löfdahl C.-G. Egesten A. (2008). Increased risk of both ulcerative colitis and Crohn's disease in a population suffering from COPD. Lung 186, 167–172. 10.1007/s00408-008-9080-z 18330638

[B36] Elgamal Z. Singh P. Geraghty P. (2021). The upper airway microbiota, environmental exposures, inflammation, and disease. Medicina 57:823. 10.3390/medicina57080823 34441029 PMC8402057

[B37] Enaud R. Prevel R. Ciarlo E. Beaufils F. Wieërs G. Guery B.. (2020). The Gut-lung axis in health and respiratory diseases: a place for inter-organ and inter-kingdom crosstalks. Front. Cell. Infect. Microbiol. 10:9. 10.3389/fcimb.2020.00009 32140452 PMC7042389

[B38] Fan J. Li X. Gao Y. Zhou J. Wang S. Huang B.. (2020). The lung tissue microbiota features of 20 deceased patients with COVID-19. J. Infect. 81, e64–e67. 10.1016/j.jinf.2020.06.047 32579991 PMC7306202

[B39] Fang G. Wang S. Chen Q. Luo H. Lian X. Shi D. (2023). Time-restricted feeding affects the fecal microbiome metabolome and its diurnal oscillations in lung cancer mice. Neoplasia 45:100943. 10.1016/j.neo.2023.100943 37852131 PMC10590998

[B40] Farber H. W. Miller D. P. Poms A. D. Badesch D. B. Frost A. E. Rouzic E. M.-L.. (2015). Five-year outcomes of patients enrolled in the REVEAL registry. Chest 148, 1043–1054. 10.1378/chest.15-0300 26066077

[B41] Fazleen A. Wilkinson T. (2020). Early COPD: current evidence for diagnosis and management. Ther. Adv. Respir. Dis. 14:175346662094212. 10.1177/1753466620942128 32664818 PMC7394029

[B42] Fukuda S. Toh H. Hase K. Oshima K. Nakanishi Y. Yoshimura K.. (2011). Bifidobacteria can protect from enteropathogenic infection through production of acetate. Nature 469, 543–547. 10.1038/nature09646 21270894

[B43] Gans M. D. Gavrilova T. (2020). Understanding the immunology of asthma: pathophysiology, biomarkers, and treatments for asthma endotypes. Paediatr. Respir. Rev. 36, 118–127. 10.1016/j.prrv.2019.08.002 31678040

[B44] Gasse P. Riteau N. Charron S. Girre S. Fick L. Pétrilli V.. (2009). Uric acid is a danger signal activating NALP3 inflammasome in lung injury inflammation and fibrosis. Am. J. Respir. Crit. Care Med. 179, 903–913. 10.1164/rccm.200808-1274OC 19218193

[B45] Gauguet S. D'Ortona S. Ahnger-Pier K. Duan B. Surana N. K. Lu R.. (2015). Intestinal microbiota of mice influences resistance to *staphylococcus aureus* pneumonia. Infect. Immun. 83, 4003–4014. 10.1128/IAI.00037-15 26216419 PMC4567647

[B46] GBD 2019 Diseases and Injuries Collaborators (2020). Global burden of 369 diseases and injuries in 204 countries and territories, 1990-2019: a systematic analysis for the Global Burden of Disease Study 2019. Lancet 396, 1204–1222. 10.1016/S0140-6736(20)30925-9 33069326 PMC7567026

[B47] Gokulan K. Joshi M. Khare S. Bartter T. (2022). Lung microbiome, gut–lung axis and chronic obstructive pulmonary disease. Curr. Opin. Pulm. Med. 28, 134–138. 10.1097/MCP.0000000000000853 34907959

[B48] Gong G. Song S. Su J. (2021). Pulmonary fibrosis alters gut microbiota and associated metabolites in mice: an integrated 16S and metabolomics analysis. Life Sci. 264:118616. 10.1016/j.lfs.2020.118616 33098825

[B49] Goto T. (2022). Microbiota and lung cancer. Semin. Cancer Biol. 86, 1–10. 10.1016/j.semcancer.2022.07.006 35882258

[B50] Gupta A. Madhavan M. V. Sehgal K. Nair N. Mahajan S. Sehrawat T. S.. (2020). Extrapulmonary manifestations of COVID-19. Nat. Med. 26, 1017–1032. 10.1038/s41591-020-0968-3 32651579 PMC11972613

[B51] Hakozaki T. Richard C. Elkrief A. Hosomi Y. Benlaïfaoui M. Mimpen I.. (2020). The Gut microbiome associates with immune checkpoint inhibition outcomes in patients with advanced non–small cell lung cancer. Cancer Immunol. Res. 8, 1243–1250. 10.1158/2326-6066.CIR-20-0196 32847937

[B52] Hayden H. S. Eng A. Pope C. E. Brittnacher M. J. Vo A. T. Weiss E. J.. (2020). Fecal dysbiosis in infants with cystic fibrosis is associated with early linear growth failure. Nat. Med. 26, 215–221. 10.1038/s41591-019-0714-x 31959989 PMC7018602

[B53] He J. Zhang P. Shen L. Niu L. Tan Y. Chen L.. (2020). Short-chain fatty acids and their association with signalling pathways in inflammation, glucose and lipid metabolism. Int. J. Mol. Sci. 21:6356. 10.3390/ijms21176356 32887215 PMC7503625

[B54] Ho K. J. Varga J. (2017). Early-life gut dysbiosis: a driver of later-life fibrosis? J. Investig. Dermatol. 137, 2253–2255. 10.1016/j.jid.2017.08.017 29055411

[B55] Hong W. Mo Q. Wang L. Peng F. Zhou Y. Zou W.. (2021). Changes in the gut microbiome and metabolome in a rat model of pulmonary arterial hypertension. Bioengineered 12, 5173–5183. 10.1080/21655979.2021.1952365 34405758 PMC8806624

[B56] Hugenholtz F. de Vos W. M. (2018). Mouse models for human intestinal microbiota research: a critical evaluation. Cell. Mol. Life Sci. 75, 149–160. 10.1007/s00018-017-2693-8 29124307 PMC5752736

[B57] Janowiak P. Szymanowska-Narloch A. Siemińska A. (2022). IPF respiratory symptoms management — current evidence. Front. Med. 9:917973. 10.3389/fmed.2022.917973 35966835 PMC9368785

[B58] Jeong D. Woo Y. D. Chung D. H. (2023). Invariant natural killer T cells in lung diseases. Exp. Mol. Med. 55, 1885–1894. 10.1038/s12276-023-01024-x 37696892 PMC10545712

[B59] Jiao J. Tang Q. Wang T. Fan J. Zhang T. Bi K.. (2022). The therapeutic effect of Xuanbai Chengqi Decoction on chronic obstructive pulmonary disease with excessive heat in the lung and fu-organs based on gut and lung microbiota as well as metabolic profiles. J. Chromatogr. B 1198:123250. 10.1016/j.jchromb.2022.123250 35421697

[B60] Juan Z. Zhao-Ling S. Ming-Hua Z. Chun W. Hai-Xia W. Meng-Yun L.. (2017). Oral administration of *Clostridium butyricum* CGMCC0313-1 reduces ovalbumin-induced allergic airway inflammation in mice. Respirology 22, 898–904. 10.1111/resp.12985 28122397

[B61] Kabel A. M. Omar M. S. Elmaaboud M. A. A. (2016). Amelioration of bleomycin-induced lung fibrosis in rats by valproic acid and butyrate: role of nuclear factor kappa-B, proinflammatory cytokines and oxidative stress. Int. Immunopharmacol. 39, 335–342. 10.1016/j.intimp.2016.08.008 27526269

[B62] Kageyama T. Ito T. Tanaka S. Nakajima H. (2024). Physiological and immunological barriers in the lung. Semin. Immunopathol. 45, 533–547. 10.1007/s00281-024-01003-y 38451292 PMC11136722

[B63] Kan H. X. Cao Y. Ma Y. Zhang Y. L. Wang J. Li J.. (2024). Efficacy and safety of probiotics, prebiotics, and synbiotics for the prevention of colorectal cancer and precancerous lesion in high-risk populations: a systematic review and meta-analysis of randomized controlled trials. J. Dig. Dis. 25, 14–26. 10.1111/1751-2980.13247 38126945

[B64] Kato A. Hulse K. E. Tan B. K. Schleimer R. P. (2013). B-lymphocyte lineage cells and the respiratory system. J. Allergy Clin. Immunol. 131, 933–957. 10.1016/j.jaci.2013.02.023 23540615 PMC3628816

[B65] Kaur R. Chupp G. (2019). Phenotypes and endotypes of adult asthma: moving toward precision medicine. J. Allergy Clin. Immunol. 144, 1–12. 10.1016/j.jaci.2019.05.031 31277742

[B66] Kim S. Rigatto K. Gazzana M. B. Knorst M. M. Richards E. M. Pepine C. J.. (2020). Altered gut microbiome profile in patients with pulmonary arterial hypertension. Hypertension 75, 1063–1071. 10.1161/HYPERTENSIONAHA.119.14294 32088998 PMC7067661

[B67] Kim S.-K. Guevarra R. B. Kim Y.-T. Kwon J. Kim H. Cho J. H.. (2019). Role of probiotics in human gut microbiome-associated diseases. J. Microbiol. Biotechnol. 29, 1335–1340. 10.4014/jmb.1906.06064 31434172

[B68] King D. E. Egan B. M. Geesey M. E. (2003). Relation of dietary fat and fiber to elevation of C-reactive protein. Am. J. Cardiol. 92, 1335–1339. 10.1016/j.amjcard.2003.08.020 14636916

[B69] Kolhe R. Sahajpal N. S. Vyavahare S. Dhanani A. S. Adusumilli S. Ananth S.. (2021). Alteration in nasopharyngeal microbiota profile in aged patients with COVID-19. Diagnostics 11:1622. 10.3390/diagnostics11091622 34573964 PMC8467337

[B70] Kristensen M. Prevaes S. M. P. J. Kalkman G. Tramper-Stranders G. A. Hasrat R. de Winter- de Groot K. M.. (2020). Development of the gut microbiota in early life: the impact of cystic fibrosis and antibiotic treatment. J. Cyst. Fibrosis 19, 553–561. 10.1016/j.jcf.2020.04.007 32487494

[B71] Labaki W. W. Han M. K. (2020). Chronic respiratory diseases: a global view. Lancet Respir. Med. 8, 531–533. 10.1016/S2213-2600(20)30157-0 32526184 PMC8034823

[B72] Lacorcia M. Bhattacharjee S. Laubhahn K. Alhamdan F. Ram M. Muschaweckh A.. (2021). Fetomaternal immune cross talk modifies T-cell priming through sustained changes to DC function. J. Allergy Clin. Immunol. 148, 843–857.e6. 10.1016/j.jaci.2021.02.031 33684437

[B73] Lau W. L. Tran T. Rhee C. M. Kalantar-Zadeh K. Vaziri N. D. (2021). Diabetes and the gut microbiome. Semin. Nephrol. 41, 104–113. 10.1016/j.semnephrol.2021.03.005 34140089

[B74] Le Poul E. Loison C. Struyf S. Springael J.-Y. Lannoy V. Decobecq M.-E.. (2003). Functional characterization of human receptors for short chain fatty acids and their role in polymorphonuclear cell activation. J. Biol. Chem. 278, 25481–25489. 10.1074/jbc.M301403200 12711604

[B75] Lee H. Y. Nam S. Kim M. J. Kim S. J. Back S. H. Yoo H. J. (2021). Butyrate prevents TGF-β1-induced alveolar myofibroblast differentiation and modulates energy metabolism. Metabolites 11:258. 10.3390/metabo11050258 33922080 PMC8143476

[B76] Li G. Lin J. Zhang C. Gao H. Lu H. Gao X.. (2021). Microbiota metabolite butyrate constrains neutrophil functions and ameliorates mucosal inflammation in inflammatory bowel disease. Gut Microbes 13:1968257. 10.1080/19490976.2021.1968257 34494943 PMC8437544

[B77] Li L. Sun Q. Xiao H. Zhang Q. Xu S. Lai L.. (2022). Aerosol inhalation of heat-killed *Clostridium butyricum* CGMCC0313-1 alleviates allergic airway inflammation in mice. J. Immunol. Res. 2022:8447603. 10.1155/2022/8447603 36033385 PMC9410851

[B78] Li M. van Esch B. C. A. M. Wagenaar G. T. M. Garssen J. Folkerts G. Henricks P. A. J. (2018). Pro- and anti-inflammatory effects of short chain fatty acids on immune and endothelial cells. Eur. J. Pharmacol. 831, 52–59. 10.1016/j.ejphar.2018.05.003 29750914

[B79] Li Y. Gao H. Zhao L. Wang J. (2022). Osteoporosis in COPD patients: risk factors and pulmonary rehabilitation. Clin. Respir. J. 16, 487–496. 10.1111/crj.13514 35688435 PMC9329018

[B80] Li Z.-J. Zhang H.-Y. Ren L.-L. Lu Q.-B. Ren X. Zhang C.-H.. (2021). Etiological and epidemiological features of acute respiratory infections in China. Nat. Commun. 12:5026. 10.1038/s41467-021-25120-6 34408158 PMC8373954

[B81] Liu F. Li J. Guan Y. Lou Y. Chen H. Xu M.. (2019). Dysbiosis of the gut microbiome is associated with tumor biomarkers in lung cancer. Int. J. Biol. Sci. 15, 2381–2392. 10.7150/ijbs.35980 31595156 PMC6775324

[B82] Liu J. D. Bayir H. O. Cosby D. E. Cox N. A. Williams S. M. Fowler J. (2017). Evaluation of encapsulated sodium butyrate on growth performance, energy digestibility, gut development, and *Salmonella colonization* in broilers. Poult. Sci. 96, 3638–3644. 10.3382/ps/pex174 28938774

[B83] Liu K. Victora G. D. Schwickert T. A. Guermonprez P. Meredith M. M. Yao K.. (2009). *In vivo* analysis of dendritic cell development and homeostasis. Science 324, 392–397. 10.1126/science.1170540 19286519 PMC2803315

[B84] Liu S. Gao J. Zhu M. Liu K. Zhang H.-L. (2020). Gut microbiota and dysbiosis in Alzheimer's disease: implications for pathogenesis and treatment. Mol. Neurobiol. 57, 5026–5043. 10.1007/s12035-020-02073-3 32829453 PMC7541367

[B85] Lopes-Pacheco M. (2020). CFTR modulators: the changing face of cystic fibrosis in the era of precision medicine. Front. Pharmacol. 10:1662. 10.3389/fphar.2019.01662 32153386 PMC7046560

[B86] Luna-López R. Ruiz Martín A. Escribano Subías P. (2022). Hipertensión arterial pulmonar. Med. Clín. 158, 622–629. 10.1016/j.medcli.2022.01.003 35279313

[B87] Lv L. Jiang H. Chen Y. Gu S. Xia J. Zhang H.. (2021). The faecal metabolome in COVID-19 patients is altered and associated with clinical features and gut microbes. Anal. Chim. Acta 1152:338267. 10.1016/j.aca.2021.338267 33648648 PMC7847702

[B88] Ma H. Wu X. Li Y. Xia Y. (2022). Research progress in the molecular mechanisms, therapeutic targets, and drug development of idiopathic pulmonary fibrosis. Front. Pharmacol. 13:963054. 10.3389/fphar.2022.963054 35935869 PMC9349351

[B89] Ma Y. Griffith J. A. Chasan-Taber L. Olendzki B. C. Jackson E. Stanek E. J.. (2006). Association between dietary fiber and serum C-reactive protein. Am. J. Clin. Nutr. 83, 760–766. 10.1093/ajcn/83.4.760 16600925 PMC1456807

[B90] Ma Y. Hébert J. R. Li W. Bertone-Johnson E. R. Olendzki B. Pagoto S. L.. (2008). Association between dietary fiber and markers of systemic inflammation in the Women's Health Initiative Observational Study. Nutrition 24, 941–949. 10.1016/j.nut.2008.04.005 18562168 PMC2603616

[B91] Mahesh S. Ramamurthy M. B. (2022). Management of acute asthma in children. Indian J. Pediatr. 89, 366–372. 10.1007/s12098-021-04051-6 35147928

[B92] Mandras S. A. Mehta H. S. Vaidya A. (2020). Pulmonary hypertension: a brief guide for clinicians. Mayo Clin. Proc. 95, 1978–1988. 10.1016/j.mayocp.2020.04.039 32861339

[B93] Manni M. L. Heinrich V. A. Buchan G. J. O'Brien J. P. Uvalle C. Cechova V.. (2021). Nitroalkene fatty acids modulate bile acid metabolism and lung function in obese asthma. Sci. Rep. 11:17788. 10.1038/s41598-021-96471-9 34493738 PMC8423735

[B94] Meeker S. M. Mears K. S. Sangwan N. Brittnacher M. J. Weiss E. J. Treuting P. M.. (2020). CFTR dysregulation drives active selection of the gut microbiome. PLoS Pathog. 16:e1008251. 10.1371/journal.ppat.1008251 31961914 PMC6994172

[B95] Mei Q. Liu Z. Zuo H. Yang Z. Qu J. (2022). Idiopathic pulmonary fibrosis: an update on pathogenesis. Front. Pharmacol. 12:797292. 10.3389/fphar.2021.797292 35126134 PMC8807692

[B96] Miller R. L. Grayson M. H. Strothman K. (2021). Advances in asthma: new understandings of asthma's natural history, risk factors, underlying mechanisms, and clinical management. J. Allergy Clin. Immunol. 148, 1430–1441. 10.1016/j.jaci.2021.10.001 34655640

[B97] Mörbe U. M. Jørgensen P. B. Fenton T. M. von Burg N. Riis L. B. Spencer J.. (2021). Human gut-associated lymphoid tissues (GALT); diversity, structure, and function. Mucosal Immunol. 14, 793–802. 10.1038/s41385-021-00389-4 33753873

[B98] Nagata N. Takeuchi T. Masuoka H. Aoki R. Ishikane M. Iwamoto N.. (2023). Human gut microbiota and its metabolites impact immune responses in COVID-19 and its complications. Gastroenterology 164, 272–288. 10.1053/j.gastro.2022.09.024 36155191 PMC9499989

[B99] O'Donnell D. E. Milne K. M. James M. D. de Torres J. P. Neder J. A. (2020). Dyspnea in COPD: new mechanistic insights and management implications. Adv. Ther. 37, 41–60. 10.1007/s12325-019-01128-9 31673990 PMC6979461

[B100] Olszak T. An D. Zeissig S. Vera M. P. Richter J. Franke A.. (2012). Microbial exposure during early life has persistent effects on natural killer T cell function. Science 336, 489–493. 10.1126/science.1219328 22442383 PMC3437652

[B101] Palafox-Carlos H. Ayala-Zavala J. F. González-Aguilar G. A. (2011). The role of dietary fiber in the bioaccessibility and bioavailability of fruit and vegetable antioxidants. J. Food Sci. 76, R6–R15. 10.1111/j.1750-3841.2010.01957.x 21535705 PMC3052441

[B102] Parada Venegas D. De La Fuente M. K. Landskron G. González M. J. Quera R. Dijkstra G.. (2019). Short chain fatty acids (SCFAs)-mediated gut epithelial and immune regulation and its relevance for inflammatory bowel diseases. Front. Immunol. 10:277. 10.3389/fimmu.2019.00277 30915065 PMC6421268

[B103] Park H. J. Jeong O.-Y. Chun S. H. Cheon Y. H. Kim M. Kim S.. (2021). Butyrate improves skin/lung fibrosis and intestinal dysbiosis in bleomycin-induced mouse models. IJMS 22:2765. 10.3390/ijms22052765 33803282 PMC7967124

[B104] Paudel D. Nair D. V. T. Tian S. Hao F. Goand U. K. Joseph G.. (2024). Dietary fiber guar gum-induced shift in gut microbiota metabolism and intestinal immune activity enhances susceptibility to colonic inflammation. Gut Microbes 16:2341457. 10.1080/19490976.2024.2341457 38630030 PMC11028019

[B105] Pleguezuelos-Manzano C. Puschhof J. Rosendahl Huber A. van Hoeck A. Wood H. M. Nomburg J.. (2020). Mutational signature in colorectal cancer caused by genotoxic pks+ E. coli. Nature 580, 269–273. 10.1038/s41586-020-2080-8 32106218 PMC8142898

[B106] Polman R. Hurst J. R. Uysal O. F. Mandal S. Linz D. Simons S. (2024). Cardiovascular disease and risk in COPD: a state of the art review. Expert Rev. Cardiovasc. Ther. 22, 177–191. 10.1080/14779072.2024.2333786 38529639

[B107] Ranjbar R. Vahdati S. N. Tavakoli S. Khodaie R. Behboudi H. (2021). Immunomodulatory roles of microbiota-derived short-chain fatty acids in bacterial infections. Biomed. Pharmacother. 141:111817. 10.1016/j.biopha.2021.111817 34126349

[B108] Ratajczak W. Rył A. Mizerski A. Walczakiewicz K. Sipak O. Laszczyńska M. (2019). Immunomodulatory potential of gut microbiome-derived short-chain fatty acids (SCFAs). Acta Biochim. Pol. 66, 1–12. 10.18388/abp.2018_2648 30831575

[B109] Roediger W. E. W. (1980). The colonic epithelium in ulcerative colitis: an energy-deficiency disease? Lancet 316, 712–715. 10.1016/S0140-6736(80)91934-0 6106826

[B110] Rusu A. Buta E. L. (2021). The development of third-generation tetracycline antibiotics and new perspectives. Pharmaceutics 13, 2085. 10.3390/pharmaceutics13122085 34959366 PMC8707899

[B111] Saeed M. A. Gribben K. C. Alam M. Lyden E. R. Hanson C. K. LeVan T. D. (2020). Association of dietary fiber on asthma, respiratory symptoms, and inflammation in the adult national health and nutrition examination survey population. Ann. Am. Thorac. Soc. 17, 1062–1068. 10.1513/AnnalsATS.201910-776OC 32369709

[B112] Salter S. J. Cox M. J. Turek E. M. Calus S. T. Cookson W. O. Moffatt M. F.. (2014). Reagent and laboratory contamination can critically impact sequence-based microbiome analyses. BMC Biol. 12:87. 10.1186/s12915-014-0087-z 25387460 PMC4228153

[B113] Salvi P. S. Cowles R. A. (2021). Butyrate and the intestinal epithelium: modulation of proliferation and inflammation in homeostasis and disease. Cells 10:1775. 10.3390/cells10071775 34359944 PMC8304699

[B114] Saura-Calixto F. (2011). Dietary fiber as a carrier of dietary antioxidants: an essential physiological function. J. Agric. Food Chem. 59, 43–49. 10.1021/jf1036596 21142013

[B115] Sencio V. Machado M. G. Trottein F. (2021). The lung–gut axis during viral respiratory infections: the impact of gut dysbiosis on secondary disease outcomes. Mucosal Immunol. 14, 296–304. 10.1038/s41385-020-00361-8 33500564 PMC7835650

[B116] Singh N. Gurav A. Sivaprakasam S. Brady E. Padia R. Shi H.. (2014). Activation of Gpr109a, receptor for niacin and the commensal metabolite butyrate, suppresses colonic inflammation and carcinogenesis. Immunity 40, 128–139. 10.1016/j.immuni.2013.12.007 24412617 PMC4305274

[B117] Sivaprakasam S. Bhutia Y. D. Yang S. Ganapathy V. (2017). Short-chain fatty acid transporters: role in colonic homeostasis. Compr. Physiol. 8, 299–314. 10.1002/cphy.c170014 29357130 PMC6019286

[B118] Stern J. Pier J. Litonjua A. A. (2020). Asthma epidemiology and risk factors. Semin. Immunopathol. 42, 5–15. 10.1007/s00281-020-00785-1 32020334

[B119] Stiemsma L. T. Arrieta M.-C. Dimitriu P. A. Cheng J. Thorson L. Lefebvre D. L.. (2016). Shifts in *Lachnospira* and *Clostridium sp*. in the 3-month stool microbiome are associated with preschool age asthma. Clin. Sci. 130, 2199–2207. 10.1042/CS20160349 27634868

[B120] Sturm E. M. Knuplez E. Marsche G. (2021). Role of short chain fatty acids and apolipoproteins in the regulation of eosinophilia-associated diseases. Int. J. Mol. Sci. 22:4377. 10.3390/ijms22094377 33922158 PMC8122716

[B121] Suster D. I. Mino-Kenudson M. (2020). Molecular pathology of primary non-small cell lung cancer. Arch. Med. Res. 51, 784–798. 10.1016/j.arcmed.2020.08.004 32873398

[B122] Tagé B. S. S. Gonzatti M. B. Vieira R. P. Keller A. C. Bortoluci K. R. Aimbire F. (2024). Three main SCFAs mitigate lung inflammation and tissue remodeling Nlrp3-dependent in murine HDM-induced neutrophilic asthma. Inflammation. 10.1007/s10753-024-01983-x. [Epub ahead of print]. 38329636

[B123] Tan J. K. Macia L. Mackay C. R. (2023). Dietary fiber and SCFAs in the regulation of mucosal immunity. J. Allergy Clin. Immunol. 151, 361–370. 10.1016/j.jaci.2022.11.007 36543697

[B124] Testa I. Crescenzi O. Esposito S. (2022). Gut dysbiosis in children with cystic fibrosis: development, features and the role of gut–lung axis on disease progression. Microorganisms 11:9. 10.3390/microorganisms11010009 36677301 PMC9865868

[B125] Thibeault C. Suttorp N. Opitz B. (2021). The microbiota in pneumonia: from protection to predisposition. Sci. Transl. Med. 13:eaba0501. 10.1126/scitranslmed.aba0501 33441423

[B126] Tian Y. Li M. Song W. Jiang R. Li Y. Q. (2019). Effects of probiotics on chemotherapy in patients with lung cancer. Oncol. Lett. 17, 2836–2848. 10.3892/ol.2019.9906 30854059 PMC6365978

[B127] Trompette A. Gollwitzer E. S. Pattaroni C. Lopez-Mejia I. C. Riva E. Pernot J.. (2018). Dietary fiber confers protection against flu by shaping Ly6c- patrolling monocyte hematopoiesis and CD8+ T cell metabolism. Immunity 48, 992–1005.e8. 10.1016/j.immuni.2018.04.022 29768180

[B128] Tulic M. K. Piche T. Verhasselt V. (2016). Lung-gut cross-talk: evidence, mechanisms and implications for the mucosal inflammatory diseases. Clin. Exp. Allergy 46, 519–528. 10.1111/cea.12723 26892389

[B129] van Iersel L. E. J. Beijers R. J. H. C. G. Gosker H. R. Schols A. M. W. J. (2022). Nutrition as a modifiable factor in the onset and progression of pulmonary function impairment in COPD: a systematic review. Nutr. Rev. 80, 1434–1444. 10.1093/nutrit/nuab077 34537848 PMC9086787

[B130] Van Nimwegen F. A. Penders J. Stobberingh E. E. Postma D. S. Koppelman G. H. Kerkhof M.. (2011). Mode and place of delivery, gastrointestinal microbiota, and their influence on asthma and atopy. J. Allergy Clin. Immunol. 128, 948–955.e3. 10.1016/j.jaci.2011.07.027 21872915

[B131] Velikova T. Snegarova V. Kukov A. Batselova H. Mihova A. Nakov R. (2021). Gastrointestinal mucosal immunity and COVID-19. WJG 27, 5047–5059. 10.3748/wjg.v27.i30.5047 34497434 PMC8384742

[B132] Verma A. Bhagchandani T. Rai A. Nikita null, Sardarni U. K. Bhavesh N. S.. (2024). Short-chain fatty acid (SCFA) as a connecting link between microbiota and gut-lung axis-a potential therapeutic intervention to improve lung health. ACS Omega 9, 14648–14671. 10.1021/acsomega.3c05846 38585101 PMC10993281

[B133] Vernocchi P. Del Chierico F. Russo A. Majo F. Rossitto M. Valerio M.. (2018). Gut microbiota signatures in cystic fibrosis: loss of host CFTR function drives the microbiota enterophenotype. PLoS ONE 1:e0208171. 10.1371/journal.pone.0208171 30521551 PMC6283533

[B134] Vogelmeier C. F. Román-Rodríguez M. Singh D. Han M. K. Rodríguez-Roisin R. Ferguson G. T. (2020). Goals of COPD treatment: focus on symptoms and exacerbations. Respir. Med. 166:105938. 10.1016/j.rmed.2020.105938 32250871

[B135] Wang J. Jiang M. Chen X. Montaner L. J. (2020). Cytokine storm and leukocyte changes in mild versus severe SARS-CoV-2 infection: review of 3939 COVID-19 patients in China and emerging pathogenesis and therapy concepts. J. Leukoc. Biol. 108, 17–41. 10.1002/JLB.3COVR0520-272R 32534467 PMC7323250

[B136] Wang Q. He Z. Zhu J. Hu M. Yang L. Yang H. (2024). Polyphyllin B inhibited STAT3/NCOA4 pathway and restored gut microbiota to ameliorate lung tissue injury in cigarette smoke-induced mice. BMC Biotechnol. 24:13. 10.1186/s12896-024-00837-6 38459479 PMC10921762

[B137] Wang Y. Leong L. E. X. Keating R. L. Kanno T. Abell G. C. J. Mobegi F. M.. (2019). Opportunistic bacteria confer the ability to ferment prebiotic starch in the adult cystic fibrosis gut. Gut Microbes 10, 367–381. 10.1080/19490976.2018.1534512 30359203 PMC6546330

[B138] Wedgwood S. Gerard K. Halloran K. Hanhauser A. Monacelli S. Warford C.. (2020). Intestinal dysbiosis and the developing lung: the role of toll-like receptor 4 in the gut-lung axis. Front. Immunol. 11:357. 10.3389/fimmu.2020.00357 32194566 PMC7066082

[B139] Whiteside S. A. McGinniss J. E. Collman R. G. (2021). The lung microbiome: progress and promise. J. Clin. Invest. 131:e150473. 10.1172/JCI150473 34338230 PMC8321564

[B140] Willers M. Viemann D. (2021). Role of the gut microbiota in airway immunity and host defense against respiratory infections. Biol. Chem. 402, 1481–1491. 10.1515/hsz-2021-0281 34599869

[B141] Williams L. M. Scott H. A. Wood L. G. (2019). Soluble fibre as a treatment for inflammation in asthma. J. Nutr. Intermed. Metab. 18:100108. 10.1016/j.jnim.2019.100108

[B142] Wu B. G. Segal L. N. (2017). Lung microbiota and its impact on the mucosal immune phenotype. Microbiol. Spectr. 5. 10.1128/microbiolspec.BAD-0005-2016 28643622 PMC5484071

[B143] Wu Y. Guo C. Tang L. Hong Z. Zhou J. Dong X.. (2020). Prolonged presence of SARS-CoV-2 viral RNA in faecal samples. Lancet Gastroenterol. Hepatol. 5, 434–435. 10.1016/S2468-1253(20)30083-2 32199469 PMC7158584

[B144] Wypych T. P. Wickramasinghe L. C. Marsland B. J. (2019). The influence of the microbiome on respiratory health. Nat. Immunol. 20, 1279–1290. 10.1038/s41590-019-0451-9 31501577

[B145] Xiao X. Cao Y. Chen H. (2018). Profiling and characterization of microRNAs responding to sodium butyrate treatment in A549 cells. J Cell. Biochem. 119, 3563–3573. 10.1002/jcb.26547 29231270

[B146] Yagi K. Huffnagle G. B. Lukacs N. W. Asai N. (2021). The lung microbiome during health and disease. IJMS 22:10872. 10.3390/ijms221910872 34639212 PMC8509400

[B147] Yang J. J. Yu D. Xiang Y.-B. Blot W. White E. Robien K.. (2020). Association of dietary fiber and yogurt consumption with lung cancer risk: a pooled analysis. JAMA Oncol. 6:e194107. 10.1001/jamaoncol.2019.4107 31647500 PMC6813596

[B148] Ye S. Wang L. Li S. Ding Q. Wang Y. Wan X.. (2022). The correlation between dysfunctional intestinal flora and pathology feature of patients with pulmonary tuberculosis. Front. Cell. Infect. Microbiol. 12:1090889. 10.3389/fcimb.2022.1090889 36619765 PMC9811264

[B149] Yeoh Y. K. Zuo T. Lui G. C.-Y. Zhang F. Liu Q. Li A. Y.. (2021). Gut microbiota composition reflects disease severity and dysfunctional immune responses in patients with COVID-19. Gut 70, 698–706. 10.1136/gutjnl-2020-323020 33431578 PMC7804842

[B150] Yip W. Hughes M. R. Li Y. Cait A. Hirst M. Mohn W. W.. (2021). Butyrate shapes immune cell fate and function in allergic asthma. Front. Immunol. 12:628453. 10.3389/fimmu.2021.628453 33659009 PMC7917140

[B151] Young R. P. Hopkins R. J. Marsland B. (2016). The gut-liver-lung axis. Modulation of the innate immune response and its possible role in chronic obstructive pulmonary disease. Am. J. Respir. Cell. Mol. Biol. 54, 161–169. 10.1165/rcmb.2015-0250PS 26473323

[B152] Yue Y. Ma W. Accorsi E. K. Ding M. Hu F. Willett W. C.. (2022). Long-term diet and risk of severe acute respiratory syndrome coronavirus 2 (SARS-CoV-2) infection and coronavirus disease 2019 (COVID-19) severity. Am. J. Clin. Nutr. 116, 1672–1681. 10.1093/ajcn/nqac219 35945354 PMC9384672

[B153] Zhan S. Li N. Liu C. Mao R. Wu D. Li T.. (2021). Intestinal fibrosis and gut microbiota: clues from other organs. Front. Microbiol. 12:694967. 10.3389/fmicb.2021.694967 34335525 PMC8322786

[B154] Zhang F. Wan Y. Zuo T. Yeoh Y. K. Liu Q. Zhang L.. (2022). Prolonged impairment of short-chain fatty acid and l-isoleucine biosynthesis in gut microbiome in patients with COVID-19. Gastroenterology 162, 548–561.e4. 10.1053/j.gastro.2021.10.013 34687739 PMC8529231

[B155] Zhang W.-Q. Zhao S.-K. Luo J.-W. Dong X.-P. Hao Y.-T. Li H.. (2018). Alterations of fecal bacterial communities in patients with lung cancer. Am. J. Transl. Res. 10, 3171–3185. 30416659 PMC6220220

[B156] Zhao J. Zhang X. Liu H. Brown M. A. Qiao S. (2019). Dietary protein and gut microbiota composition and function. Curr. Protein Pept. Sci. 20, 145–154. 10.2174/1389203719666180514145437 29756574

[B157] Zheng D. Liwinski T. Elinav E. (2020). Interaction between microbiota and immunity in health and disease. Cell Res. 30, 492–506. 10.1038/s41422-020-0332-7 32433595 PMC7264227

[B158] Zhou Y. Chen L. Sun G. Li Y. Huang R. (2019). Alterations in the gut microbiota of patients with silica-induced pulmonary fibrosis. J. Occup. Med. Toxicol. 14:5. 10.1186/s12995-019-0225-1 30867671 PMC6399897

[B159] Zhuang H. Cheng L. Wang Y. Zhang Y.-K. Zhao M.-F. Liang G.-D.. (2019). Dysbiosis of the gut microbiome in lung cancer. Front. Cell. Infect. Microbiol. 9:112. 10.3389/fcimb.2019.00112 31065547 PMC6489541

